# Mediator Kinase Inhibitor Selectivity and Activity in Colorectal Cancer

**DOI:** 10.1021/acschembio.5c00338

**Published:** 2025-07-02

**Authors:** Maria J. Ortiz-Ruiz, Olajumoke Popoola, Konstantinos Mitsopoulos, Robert Te-Poele, Rahul S. Samant, Gary Box, Will Court, Alexis De Haven Brandon, Sharon Gowan, Aurelie Mallinger, Toby Roe, Kate Swabey, Melanie Valenti, Bissan Al-Lazikani, Julian Blagg, Christina Esdar, Kai Schiemann, Dirk Wienke, Suzanne A. Eccles, Paul Workman, Paul A. Clarke

**Affiliations:** † Centre for Cancer Drug Discovery, 388505 The Institute of Cancer Research, London SM2 5NG, U.K.; ‡ 2792 The Healthcare Business of Merck KGaA, 64293 Darmstadt, Germany

## Abstract

The Mediator complex is a regulator of gene expression, influencing chromatin structure and RNA polymerase II-mediated transcription. Its activity is controlled by a protein kinase module, which includes cyclin-dependent kinases 8 and 19, that phosphorylates RNA polymerase II and transcription factors to regulate gene expression. Using orthogonal approaches combining chemical and genetic tools, we demonstrated the selectivity of our small-molecule inhibitors derived from 3,4,5-trisubstituted pyridine and 3-methyl-1*H*-pyrazolo­[3,4-*b*]­pyridine chemical series in human colorectal cell culture and tumor xenograft models. The lack of activity of our inhibitors in CDK8/19 double knockout models, with respect to molecular, proliferative, and antitumor end points, revealed their specificity and dependence on these kinases. Using our chemical probes and knockout models, we explored Mediator kinase function in human colorectal cancer cells. Phospho-proteome profiling revealed substrates enriched with transcription and chromatin regulators, while promoter reporter experiments identified transcription factor binding sites, including TCF/LEF and AP1, regulated by Mediator kinases. Additionally, altered phosphorylation of several Mediator subunits suggests a mechanism for the rapid regulation of the Mediator complex. Overall, our results demonstrate that CDK8 and CDK19 play pivotal roles in regulating gene expression associated with oncogene activation and signaling pathways. Further studies are warranted to elucidate their broader cellular roles and regulatory mechanisms. The selective inhibitors validated in this study will provide valuable tools for such mechanistic investigations into Mediator kinase functions and their potential therapeutic exploitation.

## Graphical abstract

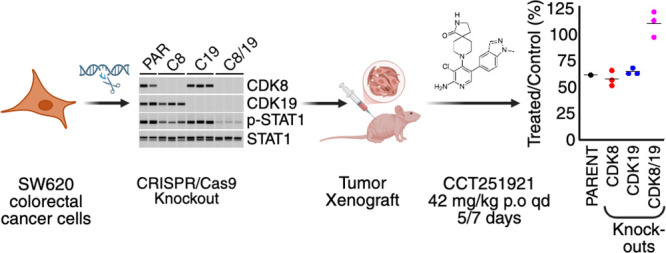

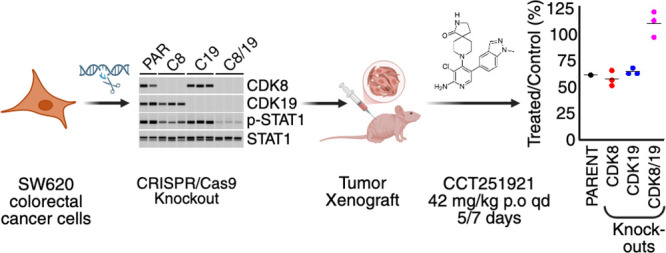

## Introduction

The Mediator is a large, evolutionarily conserved complex that influences multiple aspects of gene expression.^1–4^ The activity of the Mediator complex is regulated by the reversible association of a conserved Mediator kinase module, which comprises a cyclin-dependent protein kinase (CDK8 or CDK19), cyclin C (CCNC), MED12, and MED13.^5–7^ CDK8 and CDK19 are reported to regulate transcription by phosphorylating the C-terminal domain (CTD) of RNA polymerase II, as well as transcription factors (TFs); in the latter case altering their activity or marking them for degradation.^8–15^ CDK8 is activated by multiple stimuli and can function either as an oncogene or a tumor suppressor, depending on the cellular context.^4,16–18^ In contrast, less is known about CDK19. It has been shown to assemble into a kinase module analogous to CDK8, but can also function in a kinase-independent manner in transcriptional responses to specific stimuli.^2,5,19–21^

Understanding the cellular function of a protein kinase requires knowledge of its substrate repertoire. Substrates can be identified either through global profiling methods or on a candidate basis following perturbation using genetic or chemical approaches – strategies that have strengths and weaknesses. Genetic removal of a target kinase can result in scaffold-related deficits unrelated to the loss of kinase activity. These limitations can be overcome by use of small-molecule probe compounds, although, these too have their drawbacks, e.g., the potential for misleading off-target effects, as most biochemical and biophysical methods can confirm selective target engagement but cannot reveal the consequences or selectivity of inhibition in intact cells.^22,23^ Orthogonal approaches that combine genetic manipulation with inhibitors can help distinguish on- or off-target activity by assessing whether inhibitor activity is diminished in cells lacking the putative target. These challenges of selectivity were highlighted in studies showing that the activity of several cancer drugs in preclinical or clinical development remained unaffected by the loss of their presumed targets, indicating that these compounds may kill cells through off-target effects.^24,25^

Our primary objective was to apply a combined orthogonal approach to determine the selectivity of high-quality chemical probes derived from our two series of potent and selective small-molecule CDK8/19 inhibitors.^26–30^ Using this strategy, we verified that chemical tools from our 3,4,5-trisubstituted pyridine or 3-methyl-1*H*-pyrazolo­[3,4-*b*]­pyridine series are highly selective and potent chemical probes suitable for studying Mediator kinase function. We previously demonstrated that lead compounds from these two series were potent inhibitors of WNT signaling and inhibited growth of in vitro and in vivo models of human colorectal cancer derived from cell lines or patient biopsies.^30^ Therefore a secondary objective was to use these tool compounds, alongside genetic knockout models, to further investigate the cancer cell functions of Mediator kinases. Phospho-proteome profiling revealed enrichment in regulators of transcription and chromatin architecture, including Mediator subunits. Comparison of the single knockouts revealed that CDK8 loss had a more substantial impact on molecular readouts than CDK19 loss. However, the double CDK8/CDK19 knockout models exhibited the most pronounced alterations in molecular readouts, both in vitro and in vivo. By profiling promoter activity following inhibitor treatment in both parent and knockout cells, we identified inhibitor-responsive promoter activity associated with oncogene activation. Loss of both Mediator kinases abolished the in vivo antitumor activity of the inhibitors across multiple knockout clones, while the loss of CDK8 alone was sufficient to prevent colony growth inhibition. Overall, our data verified the selectivity of both inhibitor series for CDK8 and CDK19 and highlight their utility for probing Mediator kinase function.

## Results

### Experimental Approach, Chemical Probes, and Models

To better understand the selectivity of chemical probe compounds and the roles of CDK8 and CDK19 in human colorectal cancer models, we employed a combination of our previously characterized CDK8/19 tool compounds and CRISPR/Cas9-generated knockouts.^26–30^ The effects of these orthogonal perturbations were evaluated using phospho-proteomics, promoter reporter assays, gene expression profiling, growth assays, and solid tumor xenograft models in nude mice.

We selected active compounds and less active negative controls from our 3,4,5-trisubstituted pyridine and 3-methyl-1*H*-pyrazolo­[3,4-*b*]­pyridine chemical probe series (Figure 1A).^26–30^ The activity of our selected tool compounds was previously confirmed in CDK8 kinase activity assays, and their binding selectivity for CDK8/19 validated against other CDKs and ≈300 additional protein kinases.^27–30^ More recently the selective target occupancy of CCT251545 on CDK8/19 versus all other human CDKs was confirmed in intact cells using energy transfer probes.^31^ To substantiate the CDK8/19 selectivity of these tool compounds, we profiled their inhibitory activity in enzymatic activity assays covering 32 human CDK/cyclin pairs, representing 20 of the 21 human CDKs. All three inhibitors demonstrated the expected potent low-nanomolar IC_50_ selectivity for CDK8/cyclin C and CDK19/cyclin C and no significant activity (IC_50_ > 1 μM) against the remaining CDK/cyclin pairs in the panel, confirming their high selectivity for CDK8 and CDK19 within the CDK-family of protein kinases (Figure 1B).

**Figure 1:**
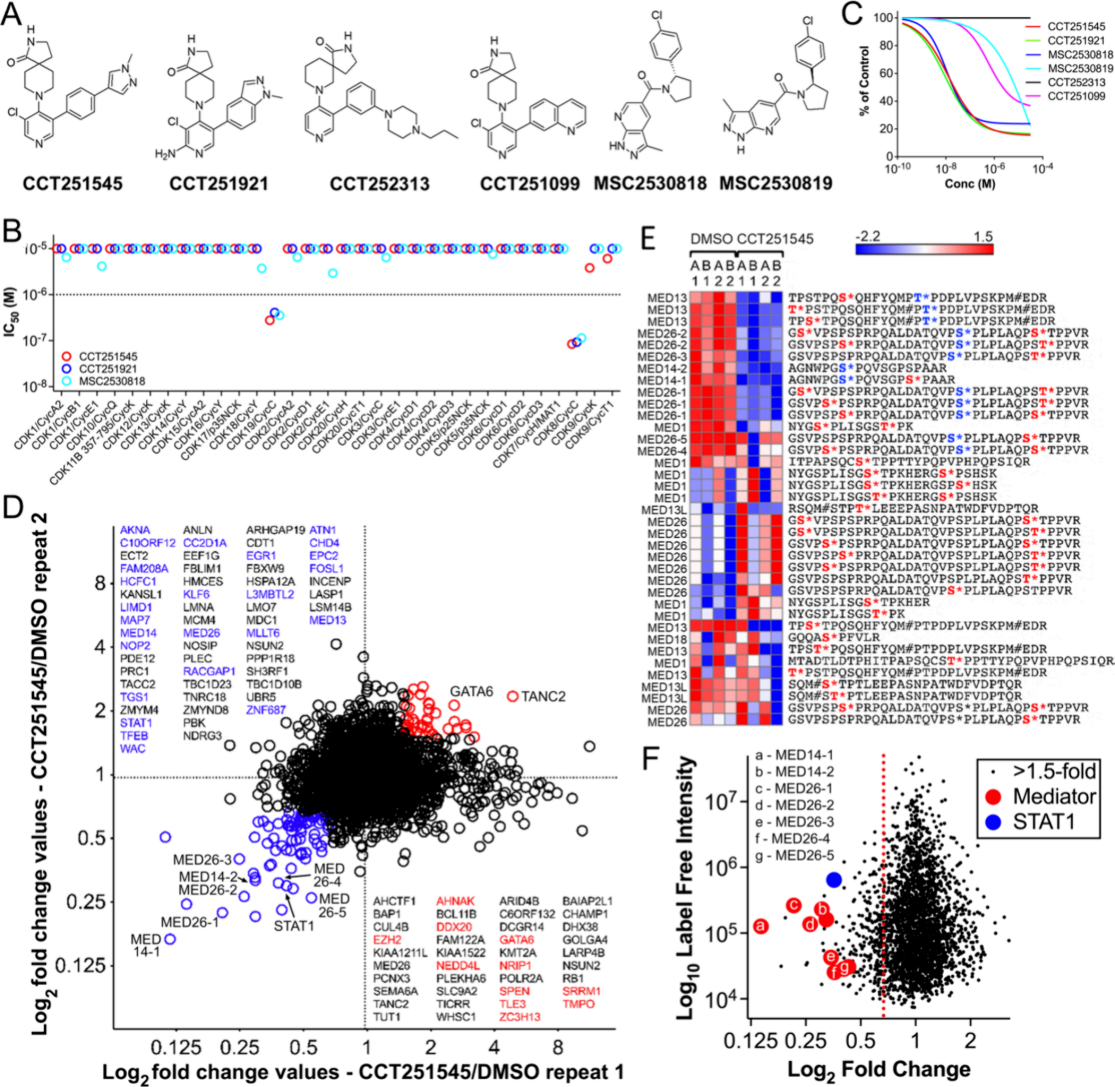
Inhibition of CDK8/19 following treatment of COLO205 human colorectal cancer cells with CCT251545 decreases phosphorylation of transcriptional regulators. (A) Chemical structures of active and less active compounds used in this study. IC_50_ values for in vitro CDK8 binding were determined using a reporter displacement assay^26^ for CCT251545 (5 nM), CCT251921 (4.9 nM), CCT251099 (103 nM), MSC2530818 (4.4 nM) and MSC2530819 (590 nM). (B) IC_50_ values were calculated for 32 human CDK/cyclin pairs using a radiometric protein kinase assay (Supporting Information and Data). The dotted line indicates a 1 μM IC_50_ threshold. (C) Linear regression plots show the response of COLO205 (*n* = 3 independent repeats) expressing a TCF/LEF WNT-reporter construct (F1756) following 6 h exposure to compound.^29^ Data are fitted using a log­(inhibitor) versus response model with a four-parameter variable slope; symbols and error bars are omitted for simplicity. (D) Plot of enriched phospho-peptides. Label-free intensities were measured in two independent biological experiments each with two technical repeats. Proteins with known links to transcription regulation are highlighted—red and blue text indicates phospho-peptides increased and decreased >1.5-fold respectively in both biological repeats after 6 h treatment with 350 nM CCT251545. (E) Hierarchical clustering of label-free intensities of Mediator subunit peptides. Red indicates increased and blue decreased intensities relative to the median. DMSO (vehicle) and CCT251545-treated samples are shown (A/B = biological repeats; 1/2 = technical repeats; * = phosphorylated residue). Predicted phospho-sites are highlighted in red, blue text indicates sites altered by CCT251545 treatment. (F) Summary plot of log_2_ fold changes of CCT251545 treatment/DMSO control versus label-free phospho-peptide intensities (red dotted line indicates 1.5-fold decrease).

Based on prior data with our chemical probes—3,4,5-trisubstituted pyridine compounds CCT251545 and CCT251921, and 3-methyl-1*H*-pyrazolo­[3,4-*b*]­pyridine compound MSC2530818—we initially used the human colorectal cancer cell line COLO205. This model is highly dependent on mutant BRAF and oncogenic β-catenin-driven WNT signaling and exhibits high basal β-catenin-dependent TCF/LEF promoter activity, which is sensitive to CDK8/19 inhibition in vitro and in vivo (Figure 1C and Supporting Figure S1A)**.** ^27,29,30^ For follow-up experiments, we used the WNT- and KRAS-dependent human colorectal cancer cell line SW620, which we have previously shown to be responsive to Mediator kinase inhibition in vitro and in vivo (Supporting Figure S1).^27,29,30^

### Transcriptional Regulators Are Enriched in Phospho-Proteome Profiles Following Mediator Kinase Inhibition by CCT251545

We phospho-proteome profiled COLO205 cells to identify Mediator kinase substrates following chemical inhibition of Mediator kinase activity. Given the confirmed CDK8/19 selectivity of our compounds in binding and enzymatic activity assays, we employed a pool of antibodies selective for the proline-rich motifs phosphorylated by the CDK family to enrich potential CDK8/19 substrates for LC-MS/MS analysis.^32^ CCT251545 demonstrated the greatest potency and selectivity in the binding and activity assays, and was used to treat COLO205 cells for 6 h at the EC_90_ for TCF/LEF promoter reporter activity (Figure 1C).^27,29–31^

In total, we identified 2,734 phospho-peptides corresponding to 1,077 individual proteins, with functions related to cell cycle, transcription, DNA damage response/repair, and adhesion (Supporting Information). The label-free intensities of 95% of the phospho-peptides remained unchanged following CCT251545 treatment. However, 123 phospho-peptides, representing 95 individual proteins, were consistently decreased by > 1.5-fold after CCT251545 treatment in both independent biological repeats (Figure 1D;Supporting Information). The validity of the profiling was supported by the observed decrease in abundance of a phospho-peptide corresponding to phospho-STAT1^SER727^ following CCT251545 exposure (Figure 1D and Supporting Figures S2A,B). The STAT1^SER727^ phospho-site, previously identified as a Mediator kinase substrate, is commonly used as a biomarker for Mediator kinase activity.^10,26–30,33–36^

The heptad repeats of the regulatory carboxy-terminal domain (CTD) of RNA polymerase II (POLR2A) are reported to be phosphorylated by multiple CDKs, including the Mediator kinases.^8,37^ We had previously found that treatment with Mediator kinase inhibitors does not affect CTD phosphorylation.^27^ Here we detected phospho-peptides corresponding to the POLR2A CTD and again observed no evidence of decreased phosphorylation following CCT251545 treatment (Supporting Figure S2C). One explanation for this discrepancy may be that only a subset of the CTD is phosphorylated by CDK8/19 and/or altered Mediator kinase-dependent CTD phosphorylation may be masked by the activity of other CDKs that also target the CTD.^38^ Similarly, CDK8/CDK7 double knockout mice maintain phosphorylation of the CTD due to activity of residual CDK7 resulting from incomplete CDK7 knockout,^39^ while CTD-phosphorylation is unaffected in CCNC knockout mouse embryo fibroblasts that lack Mediator kinase activity.^40^

Phospho-peptides corresponding to Mediator subunits Mediator subunits MED1, MED13, MED13L, MED14, MED18, and MED26 were detected, with CCT251545 treatment reducing phosphorylation at MED13^THR565^, MED14^SER1112^ and MED26^SER314^ (Figure 1D,E and Supporting Figure S2C). In the absence of phospho-specific antibodies targeting these sites, we employed immunoprecipitation using CDK-phospho-motif antibodies followed by immunoblotting with a MED14 antibody to validate the observed reduction in MED14 phosphorylation. This decrease was evident upon CCT251545 treatment but not with the inactive negative control compound, CCT251313 (Supporting Figure S2D). For MED14, the two phospho-peptides identified by phospho-proteomics both showed decreased phosphorylation in response to CCT251545 treatment (Figure 1Eand Supporting Figure S2C). In contrast, phosphorylation of MED13 and MED26 was reduced in only 2 of 5 and 5 of 10 CDK-phospho-motif peptides, respectively (Figure 1E and Supporting Figure S2C)**.** This heterogeneous phosphorylation pattern complicates validation using motif-based immunoprecipitation, as reductions at specific phospho-sites may be obscured by unchanged CDK-motif phosphorylation at other sites. Consequently, confirming these site-specific changes will require the generation of phospho-site-specific antibodies.

Functional annotation and pathway analysis revealed that 50 of the 95 identified proteins with differentially phosphorylated peptides were associated with transcriptional regulation. Additionally, substrates involved in RNA processing, DNA replication, and repair were also identified (Supporting Information). Similar findings have been reported using orthogonal proteomic profiling approaches in cortistatin A - treated HCT116 colorectal cancer cells, HEK293 genetic models, and CDK8/19 double-knockout mouse intestinal cells.^41–43^ These findings included decreased phosphorylation of Mediator complex subunits, epigenetic regulators, and transcription factors, consistent with the role of Mediator kinases in regulating transcription. Among the affected proteins, phosphorylation of STAT1^SER727^—a well-established Mediator kinase substrate—remained one of our preferred biomarkers of CDK8/19 inhibition, owing to its strong basal peptide signal and marked reduction in phosphorylation following treatment (Figure 1F). In summary, phospho-proteomic analysis following immunoprecipitation with CDK-substrate motif antibodies revealed an enrichment of transcriptional regulators among the affected proteins following CCT251545 treatment. These findings are consistent with the known role of Mediator kinases in transcriptional regulation and provide further evidence that CCT251545 is a valuable chemical tool for probing Mediator function.

### Perturbation with CDK8/19 Chemical Probes Alters the Activity of Select Promoters

We transiently transfected COLO205 cells with reporter plasmid constructs to profile gene promoter activities (Supporting Table S1) following compound treatment. Fourteen promoter reporters exhibited a >1.5-fold decrease in activity, with at least eight (TCF/LEF, cMYC, OCT4, RBP-JK/Notch, AP1, AARE, E2F, and SP1) transcription factors that bind to these promoter elements previously reported to be regulated by the Mediator kinases.^12,13,16,18,27,30,44–46^ The WNT-regulated TCF/LEF reporter construct showed the most significant decrease in activity following treatment with CCT251545 (*p*_adj_ < 0.001; Figure 2A). Further profiling of promoter reporter activity using MSC2530818,^28^ revealed overlap with the CCT251545 data, with both inhibitors reducing the activity of TCF/LEF, SP1 and PAX6 reporter constructs by more than 1.5-fold (Supporting Table S1 and Supporting Figure S3).

**Figure 2:**
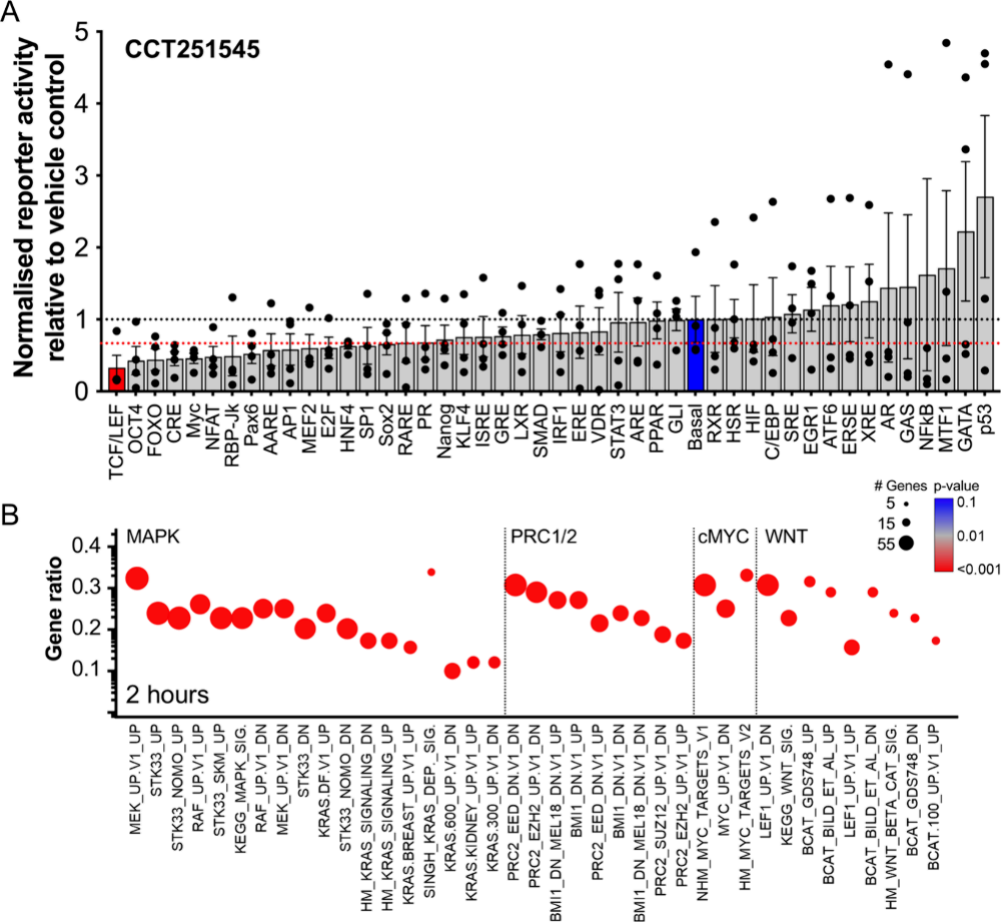
Treatment of COLO205 human colorectal cancer cells with CCT251545 alters transcription factor activity and gene expression. (A) Impact of 350 nM CCT251545 treatment for 6 h on promoter reporter activity in COLO205 cells (*n* = 4; mean ± s.e.m). The black line represents no change relative to vehicle control, while the red horizontal line highlights reporter activities repressed by >1.5-fold following treatment. The blue column indicates the basal transcriptional reporter, and red columns denote significance (*p*_adj_ < 0.001; ANOVA with Sidak’s correction). (B) GSEA of selected gene sets from microarray gene expression profiles of COLO205 cells treated with 350 nM CCT251545 for 2 h (*p*_adj_ < 0.05; Supporting Information). Symbol size is proportional to the number of altered genes in each gene set, color indicates the degree of significance and the gene ratio (*y*-axis) = number of altered genes/total genes in the gene set.

Next we incorporated our previously published microarray gene expression profiles following treatment of COLO205 cells with CCT251545 using Gene Set Enrichment Analysis (GSEA) (Figure 2B; GSE67845). Multiple gene sets associated with WNT signaling, MYC activation, polycomb repressor complex (PRC) and the RAS/RAF/MEK (MAPK) pathway were enriched following CCT251545 treatment. The inhibition of TCF/LEF promoter activity by both CCT251545 and MSC2530818, coupled with GSEA analysis showing significant enrichment of altered TCF/LEF-regulated gene expression after CCT251545 treatment, aligns with our previous observations of CDK8 and/or CDK19 knockdown in COLO205 cells that showed transcriptional activation from TCF/LEF-regulated promoters and cell survival were CDK8-dependent.^27,30^ The enrichment of gene expression downstream of RAS/RAF/MEK signaling may link to effects on the AP-1 reporter as the AP1 family of transcription factors are regulated by MAPK signaling.^47^ Overall, these findings suggest that the Mediator kinases support transcription associated with the dependency of COLO205 cells on oncogenic WNT and mutant BRAF pathways (Supporting Figure S1A).

### Effects of CRISPR/Cas9 Knockout of CDK8 and/or CDK19 on Substrate Phosphorylation; Differential Requirements for the Mediator Kinases In Vitro

To further investigate the individual roles of CDK8 and CDK19 and the selectivity of our chemical probes, we employed a CRISPR/Cas9 genetic knockout approach. The semi-adherent growth characteristics of COLO205 cells made isolating individual clones challenging. Therefore, we focused on SW620 cells, which we have previously demonstrated to be responsive to CDK8/19 inhibitors in vitro and as human tumor xenografts in vivo.^27,30^

We isolated multiple independent single and double knockout clones of CDK8 and/or CDK19, confirmed by immunoassay (Figure 3A and Supporting Figure S4A). Analysis of these clones revealed that knockout of CDK19 had no discernible effect on STAT1^SER727^ or MED14 phosphorylation compared to parental SW620 cells (Figure 3A,B and Supporting Figure S4A). In contrast, CDK8 knockout led to reduced phosphorylation of both STAT1^SER727^ and MED14. These phosphorylation events were almost completely abrogated in double knockout clones lacking both Mediator kinases (Figure 3A,B and Supporting Figure S4A). There was no evidence for compensatory altered expression of CDK19 in the single CDK8 knockout clones, or vice versa. MED12 expression was unchanged, but the expression of other kinase module proteins, MED13 and CCNC, were affected by CDK8 loss - MED13 expression was induced, whereas CCNC expression was reduced (Figure 3A). Similar to STAT1^SER727^ phosphorylation, the changes in MED13 and CCNC protein expression were absent in the CDK19 knockout but increased in the double knockout clones compared to the CDK8 knockout clones (Figure 3A). Consistent with our previously published findings from inhibitor treatments and phospho-proteome data described earlier herein, we did not observe significantly altered phosphorylation of RNA pol II CTD^SER2/5^ or E2F1^SER375^ (Supporting Figures S4B and S2C).^27^

**Figure 3:**
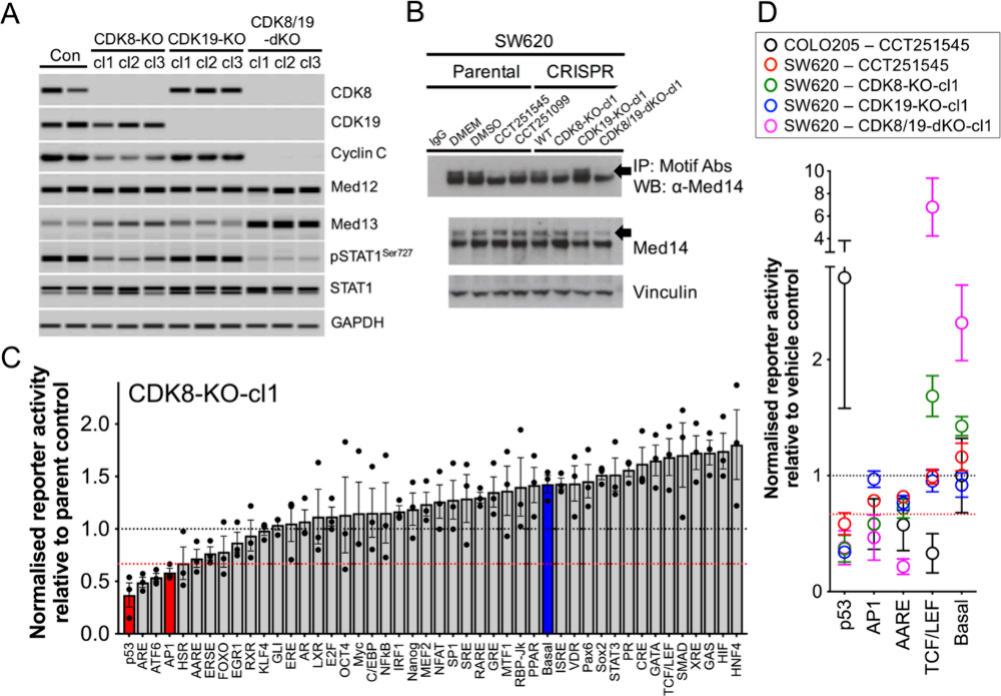
Mediator kinase knockout alters STAT1^SER727^phosphorylation and promoter activity in SW620 human colon cancer cells. (A) Capillary immunoassay of Mediator kinase module expression and pSTAT1^SER727^ biomarker in multiple independent knockout clones. GAPDH was included as a loading control. (B) CDK-substrate motif immunoprecipitation from parent SW620 cells treated for 6 h with 350 nM CCT251545, the less active control (CCT251099), or untreated knockout clones (top gel panel). Controls included IgG alone for background, DMEM for untreated cells, and DMSO as a vehicle control. MED14 was detected as a doublet pair of bands by immunoblotting of cell lysates (middle gel panel). The upper band (indicated by an arrow) is reduced by active compound treatment or CDK8 knockout. The lower panel includes vinculin as a loading control. (C) Promoter reporter results from CDK8 knockout clone 1 relative to parent SW620 cells. Data represent mean ± s.e.m (*n* = 3). The black horizontal line indicates unchanged reporter activity, while the horizontal red line highlights reporters with altered activity ±1.5-fold. The blue column represents basal transcriptional reporter activity, and the red column indicates significance (*p*_adj_ < 0.001; ANOVA with Sidak’s correction). (D) Summary of promoter reporter assays showing significance in one of the assayed conditions (>1.5 = fold, *p*_adj_ < 0.001; Figure 2A and Supporting Figure S5) from COLO205 and SW620 cells treated with CCT251545 (relative to vehicle control) or SW620 knockout clones (relative to parent SW620).

Overall. For the two biomarkers analyzed here these data demonstrate that CDK8 accounts for the majority of the individual Mediator kinase activity in SW620 cells.

### CRISPR/Cas9 Knockout of the Mediator Kinases Inhibits Promoter Reporter Activity and Gene Expression

We next examined the impact of CDK8 and/or CDK19 knockout on transcription factor activity in SW620 cells using the panel of promoter reporter constructs. Knockout of CDK8, and particularly the CDK8/19 double knockout, reduced basal activity from the CMV promoter of the transfection control plasmid, suggesting that Mediator kinase activity is required for basal CMV promoter activity. As a result, the normalized reporter data from the double knockout CRISPR clone showed an increased signal distribution relative to the parent cells, leading to an apparent increase in activity for many of the promoter reporters. For example, the basal reporters exhibited a 1.4 ± 0.1-fold and 2.3 ± 0.4-fold increase in the CDK8 knockout and CDK8/19 double knockout clones, respectively (Figure 3C, Supporting Figure S5A,B). Nevertheless, we detected significantly repressed transcription (>1.5-fold. *p*_adj_ < 0.001) from the p53 and AP1 reporters in the CDK8-KO-cl1 knockout model. There were no significant alterations in promoter activity in CDK19-KO-cl1, although similar to CDK8-KO-cl1, the p53 reporter exhibited the greatest decrease in activity (0.34 ± 0.06 s.e.m relative to the parent, *p*_adj_ = 0.0543). The loss of both Mediator kinases significantly repressed transcription from the AARE and AP1 reporters (1.5-fold, *p*_adj_ < 0.001) and also resulted in a nonsignificant decrease in p53 reporter activity (0.38 ± 0.18 s.e.m relative to the parent) (Figure 3C and Supporting Figure S5A). The CCT251545-treated parent cells showed a significant decrease in p53 reporter activity and similar to the CDK8-KO-cl1 clone some evidence for decreased activity of the AP1 (0.78 ± 0.04 s.e.m) and AARE (0.82 ± 0.06 s.e.m) promoters that were significantly altered in the CDK8-KO-cl1 and CDK8/19-dKO-cl1 clones (Supporting Figure S5C and 3D).

Overall, the comparison of inhibitor-treated parent COLO205 and SW620 cell lines with CRISPR knockouts in the promoter assay revealed distinct effects. Loss of TCF/LEF and p53 reporter activity was unique to COLO205 and SW620 cells, respectively (Figures 1C, 3C,D and Supporting Figures S4, S5). However, reduced AP1 and AARE (bound by ATF family members; Supporting Table S1) promoter reporter activity was observed in both cell lines and was significantly associated with CDK8 loss in the single or double knockouts. In addition, phospho-proteomic analysis revealed reduced phosphorylation of FOSL1, an AP1 factor, in CCT251545-treated COLO205 cells (Figure 1 and Supporting Information).

### Inhibition of CDK8, but not CDK19, Mediates Loss of Colony or Nonadherent Growth Following Treatment with Mediator Kinase Chemical Probes

We found that the in vitro cell growth rates of single and double CDK8/19 knockout SW620 cells were comparable to those of the parental line when assessed using a short-term 4-day adherent tissue culture assay (Supporting Figure S6A). However, in 14-day adherent clonogenic assays or nonadherent spheroid assays, the double CDK8/19 knockout clones exhibited ≈ 25–50% reduced growth or cloning efficiency compared to the parental cells or single knockout clones (Figure 4A and Supporting Figure S7A).

**Figure 4:**
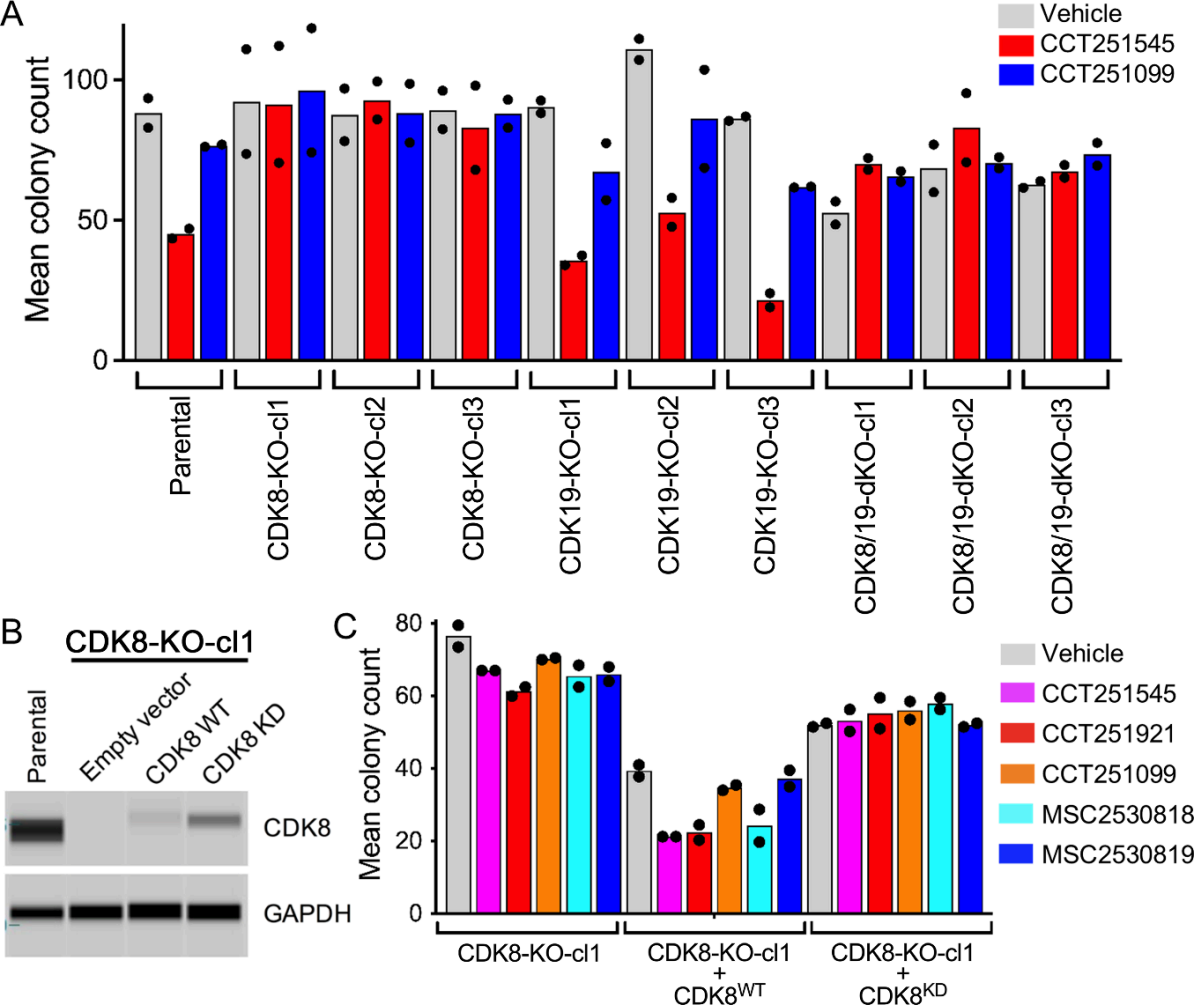
CDK8 activity is required for inhibition of colony growth of SW620 human colorectal cancer cells by Mediator kinase chemical probes. (A) Colony assays of parent SW620 cells or three individual clones per knockout. The cells were treated with 350 nM CCT251545 or CCT251099, a structurally related less active control compound, for 10 days (*n* = 2 independent repeats). (B) Immunoblot of CDK8 expression for CDK8 knockout clone CDK8-KO-cl1 transfected with an empty vector, wild-type CDK8 (CDK8^WT^), or the kinase-inactive CDK8^D173A^ (CDK8^KD^) mutant. GAPDH was included as a loading control. (C) Transfected cells from (B) were treated for 10 days with 350 nM of either CCT251545, CCT251921 (from the 3,4,5-trisubstituted pyridine series), or MSC2530818 (from the 3-methyl-1*H*-pyrazolo­[3,4-*b*]­pyridine series). Structurally related but less active controls, CCT251099 and MSC2530819, were included for comparison (*n* = 2).

To further compare the effects of genetic knockout versus chemical inhibition of Mediator kinase activity, we evaluated the growth response of the knockout clones to compound treatment. Previously, we demonstrated that parent SW620 cells are sensitive to treatment with the 3,4,5-trisubstituted pyridine series (CCT251545 and CCT251921) and the 3-methyl-1*H*-pyrazolo­[3,4-*b*]­pyridine series (MSC2530818) under both adherent colony and soft agar colony growth conditions.^27,30^ Colony growth of parent cells and single CDK19 knockout clones was similarly inhibited by both series of dual CDK8/19 tool compounds but not by structurally related, much less active control compounds (Figure 4A and Supporting Figure S6B,C). In contrast, the colony growth inhibitory activity of both compound series was reduced or completely lost in single CDK8 or double CDK8/19 knockout clones. Finally, when cells were grown as spheroids and treated with CCT251545, CDK19 knockout clones showed growth inhibition similar to the parental line, whereas CDK8 and double knockout clones were again insensitive to treatment (Supporting Figure S7B).

To investigate the requirement for CDK8 protein kinase catalytic activity in mediating the effects of compounds in the colony growth assay, we stably restored expression of either wild-type CDK8 or the kinase-inactive CDK8^D173A^ mutant in a representative CDK8 knockout clone.^27^ Expression of the active or inactive CDK8 constructs was lower than the parental CDK8 expression and their cloning efficiency was reduced. Despite this we found that only re-expression of the active wild-type CDK8, and not the kinase dead CDK8, could restore sensitivity to compounds from both chemical series. This sensitivity was not observed following treatment with the corresponding inactive control compounds (Figure 4B,C). These findings indicate that inhibition of CDK8 kinase activity is critical for the colony growth inhibition of SW620 cells by chemical probes from the 3,4,5-trisubstituted pyridine or 3-methyl-1*H*-pyrazolo­[3,4-*b*]­pyridine series.

### CDK8 and 19 Are Required for the In Vivo Molecular Response to CCT251921 Treatment of Tumor Xenografts

We next used the genetic models to examine the effects of CDK8 and/or CDK19 knockout on in vivo solid tumor xenograft growth in mice. Our findings showed that Mediator kinase loss had no significant impact on tumor establishment or growth rates in xenografts derived from two independent clones for each of the single or dual knockouts (Figure 5A). Consistent with our earlier in vitro observations (Figures 3A and Supporting Figure S4A), STAT1^SER727^ phosphorylation was unaffected by CDK19 loss but was significantly reduced in CDK8 knockout tumors (*p* < 0.0001) and almost entirely abrogated in tumors lacking both kinases (*p* < 0.0001; Figure 5B).

**Figure 5:**
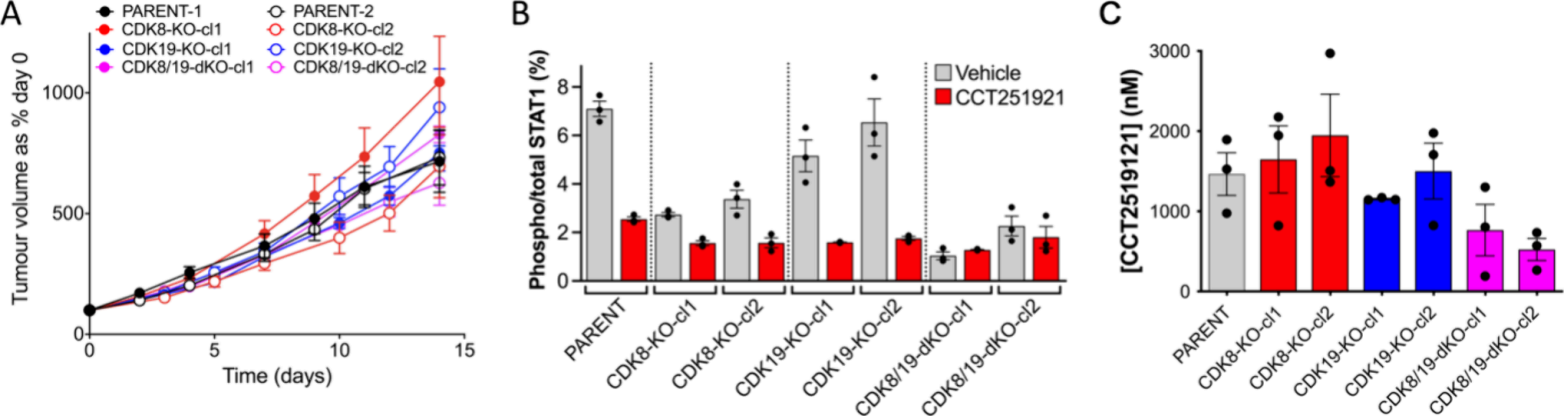
Growth of untreated SW620 human colorectal cancer tumor xenografts and pharmacokinetics of CCT251921. (A) Growth of untreated parent SW620 or CRISPR/Cas9 knockout xenografts. No significant difference in growth rates between parent and knockouts were observed. (B and C) SW620 parental or knockout clones were grown as subcutaneous solid tumor xenografts in NCRI nude mice and treated for 3 days with CCT251921 (42 mg/kg p.o. qd). Plasma and tumors were collected 6 h after the final dose. (B) Phospho-STAT1^SER727^ levels relative to total STAT1 protein were quantified in tumors using an electrochemiluminescent ELISA (mean ± s.e.m). An ordinary one-way ANOVA analysis with Dunnett’s correction showed significant differences for basal levels in the parent verses the CDK8 and CDK8/19 clones (all *p* < 0.0001) and less significance for CDK19cl (*p* < 0.05) and CDK19-KO-cl2 (*p* = ns). Treatment of the parent cells and CDK19 clones significantly reduced phospho-STAT1^SER727^ levels (*p* < 0.0001), in contrast the CDK8 and CDK8/19 knockouts showed no significant change, except for CDK8-KO-cl2 (*p* < 0.05). (C) Plasma concentrations of CCT251921, an ordinary one-way ANOVA found no significant difference between plasma levels in parent and individual knockout clones.

We next examined the molecular response to 3 days treatment with CCT251921 (42 mg/kg p.o. q.d), a 3,4,5-trisubstituted pyridine derivative related to CCT251545 but with improved in vivo pharmacokinetic properties in mice (Figure 1A).^26^ Pharmacokinetic analysis of total plasma levels of CCT251921 confirmed that single and double CDK8/19 knockout tumor xenografts were exposed to plasma concentrations comparable to those observed in parental SW620 xenografts (Figure 5C). Similar to the in vitro molecular biomarker data, the tumor STAT1^SER727^ phosphorylation data indicate a partial redundancy between CDK8 and CDK19 loss, with CDK8 inhibition playing a greater role than CDK19 in the molecular response to CCT251921 treatment in vivo.

### CDK8/19 Inhibitor CCT251921 Lacks Antitumor Activity in CDK8/19 Double Knockout Cells

Finally, we evaluated the antitumor activity of the CDK8/19 inhibitor CCT251921 in vivo in xenografts of parental SW620 cells and three independent clones from each single or dual knockout. CCT251921 demonstrated similar antitumor activity across tumors derived from CDK8 (treated/control, T/C: 59 ± 6%; s.e.m.), CDK19 (T/C: 66 ± 3%) knockouts and parental cells (T/C: 63%) (Figure 6A). Interestingly, tumors from CDK8 single knockout clones retained responsiveness to CCT251921, contrasting with in vitro adherent colony growth experiments, which showed sole dependence on CDK8. In contrast to the single knockouts, double knockout clones were completely unresponsive to CCT251921 treatment (T/C: 113 ± 10%, *p* < 0.005; Figure 6A), demonstrating that inhibition of both CDK8 and CDK19 is required to slow or arrest growth in the SW620 colon cancer xenograft model.

**Figure 6:**
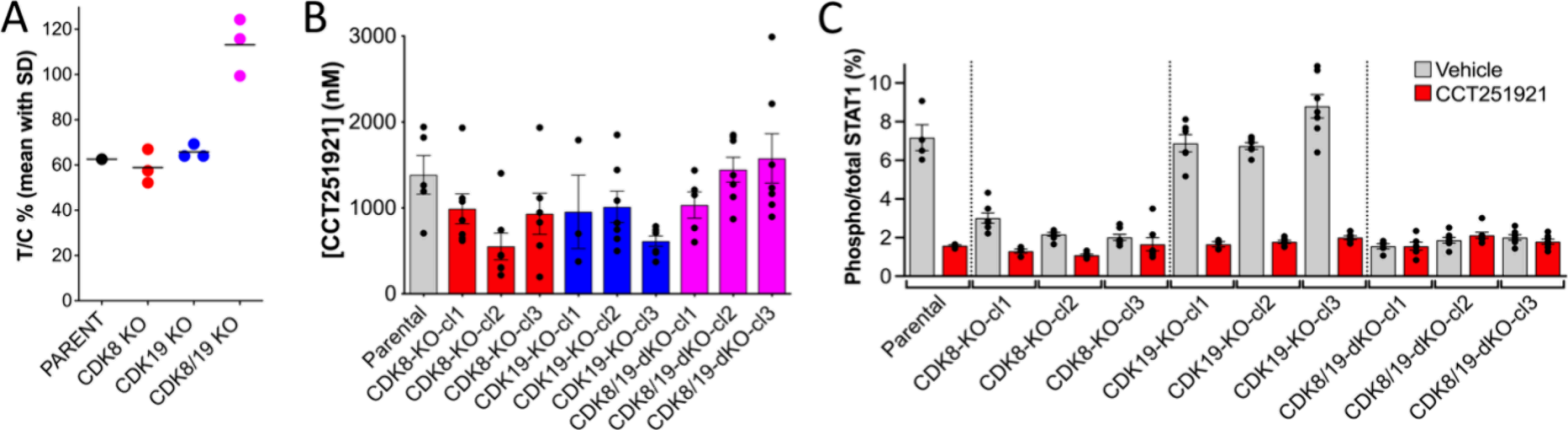
Loss of both CDK8 and 19 abrogates the antitumor activity of Mediator kinase chemical probe CCT251921 in SW620 human solid tumor xenografts. Parental SW620 cells or knockout clones were grown as tumor xenografts in NCRI nude mice and treated for 14–20 days with CCT251921 (42 mg/kg p.o qd) for 5/7 days. Three separate clones were run for each knockdown condition (*n* = 7 control or treated mice per group for the parent and each individual clone). Tumors and plasma were collected 6 h after the final dose. For all graphs an ordinary one-way ANOVA with Dunnett’s correction was run to determine significance. (A) Summary graph of CCT251921 antitumor activity. Each point represents data from a knockout clone. The parent and each of the three CDK8/19 knockout clones showed a significant difference in antitumor response (*p* < 0.005). (B) Plasma total concentrations of CCT251921 (mean ± s.e.m). With the exception of CDK8-KO-cl2 (*p* < 0.05), there were no significant differences in CCT251921 plasma levels between the parent and knockout clones. (C) Quantification of tumor phospho-STAT1^SER727^ relative to total STAT1 protein following vehicle or CCT251921 treatment (mean ± s.e.m). All CDK8 and CDK8/19 clones had significantly reduced phospho-STAT1^SER727^ (*p* < 0.0001). Treatment with CCT251921 significantly reduced phospho-STAT1^SER727^ in the parent, CDK19 (all *p* < 0.0001) and CDK8 clones (CDK8-KO-cl1 *p* < 0.001 and CDK8-KO-cl2 *p* < 0.05), but not the CDK8/19 clones.

This differential impact of CDK8 loss on response to inhibitor treatment between the in vitro and in vivo experiments is unlikely to be due to differential compound exposure, as pharmacokinetic and pharmacodynamic biomarker analyses 6 h after the final dose showed comparable exposure and STAT1^SER727^ biomarker responses (Figures 5B,C and 6B,C). Similar to data from the 3 day exposure experiment, tumors from CDK8 knockout clones exhibited an 80–90% decrease in STAT1^SER727^ phosphorylation measured at the end of this therapy experiment, which was further reduced by CCT251921 treatment (Figures 5B and 6C). Double knockout clones showed basal STAT1^SER727^ phosphorylation levels that were already reduced to CCT251921-treated levels and were unaffected by further treatment (Figure 6C).

Overall, these findings suggest that inhibition of CDK19 accounts for approximately a quarter of the inhibitable Mediator kinase activity, at least for the STAT1^SER727^ biomarker, and CDK19 inhibition contributes to the in vivo antitumor activity of CCT251921, unlike the results seen for in vitro colony growth conditions. Most importantly, absence of compound activity in the molecular and cellular end points in the double knockout clones confirms that the in vivo and antitumor effects of the 3,4,5-trisubstituted pyridine compound CCT251921 are mediated specifically through CDK8 and CDK19.

## Discussion

Recent studies highlight the complementary role of genetic approaches in preclinical drug characterization.^24,25^ Here, we combined genetic tools and chemical probes to first assess the selectivity of our tool compounds and subsequently the roles of Mediator kinases in colon cancer models.^27–29^ We hypothesized that on-target effects of our small-molecule inhibitors would be evident in parent cell lines but absent in CDK8/CDK19 knockouts, while off-target effects would impact both parent cells and knockout clones equally. To test this hypothesis, we generated multiple single and double genetic knockout clones of the Mediator kinases in SW620 human colorectal cancer cells, a model we previously demonstrated to be responsive to Mediator kinase inhibitors in vitro and in vivo*.* ^27,30^

Redundant functions of CDK8 and CDK19 have been reported, particularly in relation STAT1^SER727^ phosphorylation and gene expression.^39,42,43,48^ While some studies emphasize distinct effects between CDK8 or CDK19 loss and targeted kinase inhibition, others illustrate overlapping roles in gene expression. For instance, p53- and interferon-gamma-responsive gene expression are regulated through kinase-independent scaffolding by CDK19 and kinase-dependent mechanisms by CDK8.^19,20^ Our findings demonstrate single knockout of CDK8 has more impact than CDK19 knockout, but also that dual knockout of CDK8 and CDK19 leads to more pronounced alterations in STAT1-related signaling both in vitro and in vivo compared to single knockouts. Biomarker analysis of phospho-STAT1^SER727^ and phospho-MED14^SER1112^ in vitro revealed that CDK8 generally accounts for the majority (≈75%) of the total inhibitable Mediator kinase activity in SW620 cells. This dependency was mirrored in vivo, where residual STAT1^SER727^ phosphorylation remained sensitive to inhibition, implicating CDK19 in contributing the remaining ∼25% of activity. Interestingly, a discrepancy emerged between in vitro and in vivo data: although CDK8-knockout clones were resistant to growth suppression in vitro, tumor xenografts derived from the same clones remained responsive to inhibitor treatment in vivo. In the in vitro setting, we observed no compensatory changes in the expression of mediator kinases in the single knockout clones. However, as we did not assess their expression in the in vivo tumor xenografts, we cannot exclude the possibility of compensatory upregulation of CDK19 in the CDK8-knockout tumors. Nonetheless, our findings support the hypothesis that functional redundancy between CDK8 and CDK19 may be enhanced under tumor growth conditions, enabling CDK19 to partially compensate for the absence of CDK8 in the in vivo context (summarized in Table 1).

**Table 1:**
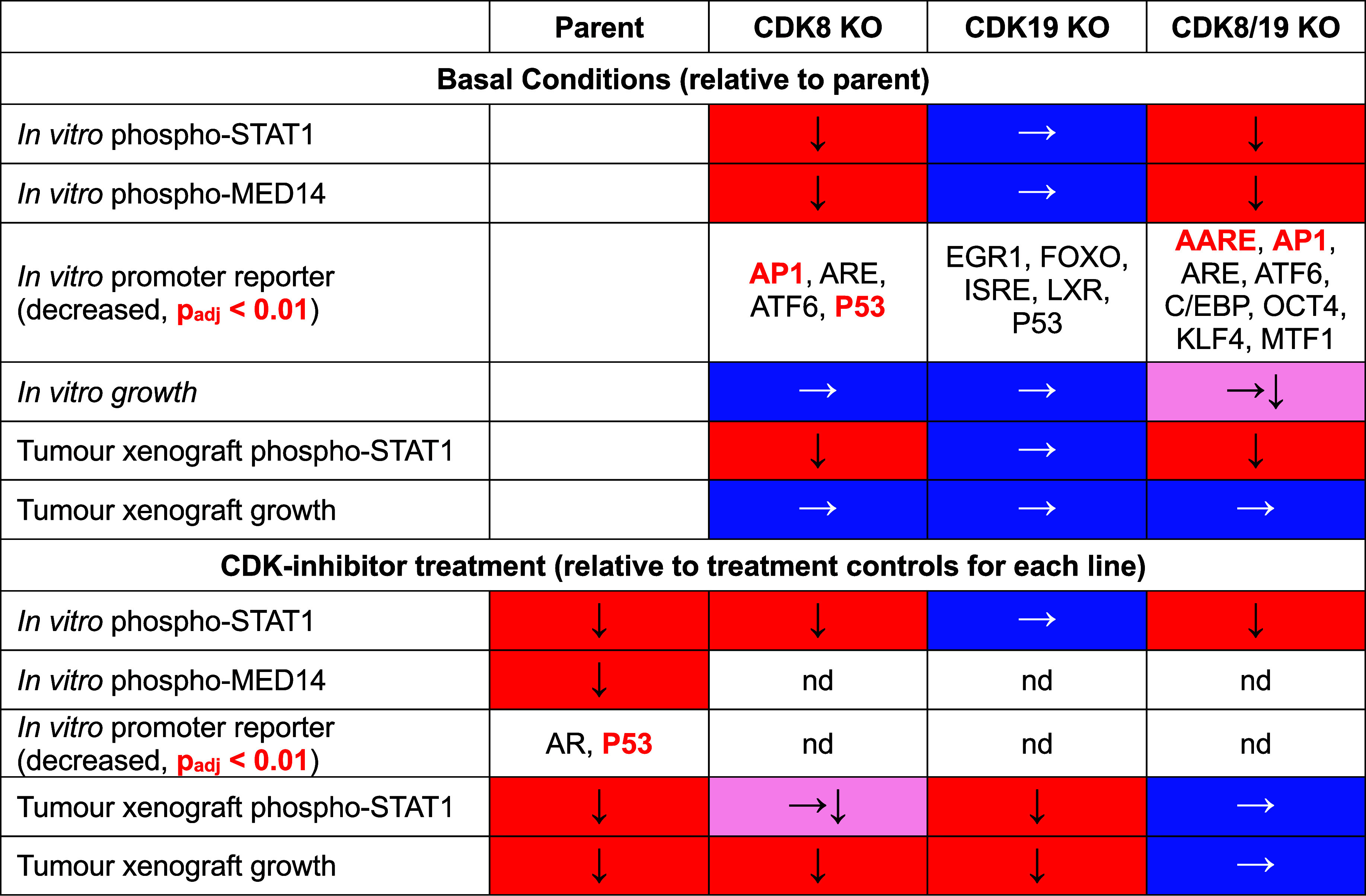
Summary of In Vitro and In Vivo Data from Basal and Compound Treatment Experiments for Parent and Mediator Kinase Knockout Lines^a^

a Blue filled cells with a white arrow indicate conditions that remained unchanged compared to the parent line under untreated conditions, or compared to their respective untreated controls in compound treatment experiments. Red-filled cells with black downward arrows indicate results that were decreased relative to the parent line (in untreated conditions) or to their untreated controls (in compound-treated conditions). Pink-filled cells denote minor changes. For promoter reporter data, black text indicates a decrease in promoter activity of more than 1.5-fold relative to control conditions. Red text denotes a statistically significant decrease in promoter activity (adjusted *p*-value < 0.01). nd = not determined.

Overall, our data confirm that our chemical tool compounds tested here act predominantly through inhibition of CDK8 kinase activity in SW620 cells, with minimal off-target effects. The absence of inhibitor activity in CDK8/19 double knockouts across all end points confirms their high selectivity. The combination of compound treatment with CRISPR-mediated gene knockout has previously been employed to reveal off-target activities of compounds.^24,25^ To our knowledge, this study provides the first example of using the present approach to explicitly demonstrate on-target selectivity for the two Mediator kinases paralogues.

Our secondary objective was to leverage the orthogonal approaches of chemical probes and genetic technology to explore Mediator kinase function in cancer cells. We profiled the phospho-proteome of COLO205 cells treated with CCT251545, our tool compound from the 3,4,5-trisubstituted pyridine series that is now listed as a high quality chemical probe on the Chemical Probes Portal (https://www.chemicalprobes.org/cct251545). We found enrichment of transcriptional regulators including decreased phosphorylation of Mediator subunits MED13, MED14, and MED26. Orthogonal mass-spectrometry approaches reported similarly decreased phosphorylation in HEK293 mediator kinase knockout models, HCT116 colon cancer cells treated with cortistatin A and mouse intestinal epithelium lacking CDK8 and CDK19.^41–43^

The activity of the Mediator kinases is crucial for driving rapid changes in stimulus-responsive gene expression. Emerging,^2,49,50^ evidence suggests that the reversible binding of the kinase module acts as a regulatory switch to control RNA polymerase binding and transcription initiation.^1,2,4,6,7,49,50^ Structural and biochemical studies indicate that MED14 facilitates Mediator complex rearrangements for RNA polymerase II recruitment.^2,51,52^ MED13 anchors the kinase module via its intrinsically disordered region (IDR),^49,50^ while MED26 is essential for overcoming RNA polymerase II recruitment blocks, but its binding is mutually exclusive with the kinase module.^5,49,50,53,54^ The IDRs of MED13 and MED26 are proposed to compete for the same surfaces on the core Mediator and recruitment of the RNA polymerase II via its CTD requires MED26-induced displacement of MED13.^49,50^ Interestingly, the MED13^THR565^ and MED26^SER314^ sites we detected fall within the IDRs of their respective proteins. The potential regulation of these subunits by CDK8 supports an appealing hypothesis: that post-translational modifications of key Mediator subunits, via CDK8 may facilitate rapid and reversible regulation of kinase module association or dissociation from the Mediator complex, thereby enabling dynamic control of Mediator-dependent transcription.

CDK8 was initially identified as an oncogene required for β-catenin-induced transcription in colorectal cancer and later implicated in regulating the AP1 family of transcription factors downstream of RAS/RAF/MEK signaling.^11,18,45,47^ Consistent with these roles in colorectal cancer, GSEA confirmed CDK8’s role in oncogenic pathways, showing inhibition of β-catenin/TCF, KRAS/MAPK, and ATF target genes following inhibitor treatment or loss of both Mediator kinases in colorectal cancer cells dependent on oncogenic beta-catenin and KRAS/BRAF signaling. Interestingly, a recent study has shown that dual CDK8 and MEK inhibition is more effective than single-agent treatments in RAS mutant cell lines.^55^ Downstream of RAS, AP1 family members can not only act as oncogenes but also as tumor suppressors.^47^ Recent findings in melanoma show that the state of the AP-1 network defines cellular plasticity inducing a diversity of differentiation states and adaptive responses to MAPK-pathway inhibitors.^56^ These and our own observations herein suggest a testable hypothesis that the reported different contextual dual oncogenic or tumor suppressor activities of CDK8 could be linked to its regulation of the AP-1 family.^16^ This could include under certain circumstances or contexts playing a critical role in transcription linked to the activation of oncogene-associated pathways. In our study, these pathways predominantly encompassed oncogenic beta-catenin and KRAS/BRAF signaling.

Our findings, alongside others, underscore the need for further investigation to fully elucidate the mechanisms used by the Mediator kinases to regulate transcription.^1–4,41,49,50^ The phosphorylation of three regulatory Mediator subunits is particularly intriguing, as it suggests mechanisms for rapid regulation by post-translation modification and warrants further exploration. Additionally, we identified potential substrates linked to RNA processing and modification, DNA replication and repair, and protein ubiquitination suggesting broader functional roles beyond transcription that merit follow-up studies. Our data also indicate that Mediator kinases may play critical roles in regulating transcription associated with the activation of oncogenic pathways and could particularly influence cancer cell plasticity and drug resistance.^55^ Importantly, the absence of CDK8/19 inhibitory activity in CDK8/19 double knockouts across molecular, proliferative, and antitumor end points in vitro and in vivo validates our 3,4,5-trisubstituted pyridine and 3-methyl-1*H*-pyrazolo­[3,4-*b*]­pyridine tool compounds as highly selective chemical probes suitable for exploring the role of the Mediator kinases in regulating gene expression in a variety of biological and disease models.

## Materials and Methods

Detailed materials and methods are available as Supporting Information **.**

### CDK8/19 Inhibitors

All compounds (Figure 1A) were synthesized and purified by published methods as described previously.^26,28,29^

### Cell Culture and Reporter Assays

Human colorectal cancer cell lines COLO205 (RRID:CVCL_0218) and SW620 (RRID:CVCL_0547) were obtained from ATCC (LGC Promochem, UK), confirmed as mycoplasma free and were authenticated by short tandem repeat DNA profiling. Drug effect was determined by alamar blue staining, clonogenic assay or WNT-pathway activity in cells carrying a TCF/LEF promoter reporter.^27,29,30^

CDK8/19 knockout SW620 cells were generated using a CRISPR/Cas9 knockout strategy with plasmid constructs expressing guide RNAs and a green or red fluorescent protein. Cells were harvested 72 h post-transfection. The top 1% fluorochrome-positive cells were isolated by FACS and colony purified. Knockout clones were identified by capillary immunoassay for CDK8 or CDK19 expression. Stable CDK8 expressing cells were established in a CDK8 knockout clone using a pCMV6-CDK8^WT^-cMyc-FLAG or construct or an inactive kinase mutant pCMV6-CDK8^D173A^-cMyc-FLAG construct.^57^

Transcription factor activity was assayed in cell lines using 45 different plasmid reporter constructs encoding firefly luciferase under the control of a basal promoter element downstream of specific transcriptional response elements (Qiagen, Germany; Supporting Table S1). Firefly luciferase under the control of basal promoter element alone was used to establish the baseline signal and cotransfection of a construct expressing renilla luciferase from a CMV-promoter was used as a transfection control. Cells were reverse-transfected with reporter plasmids, compounds were added 42 h post transfection and reporter activity quantified following 6 h continuous exposure.

### Tumour Xenograft Studies

The establishment, treatment, pharmacokinetic analyses and processing of tumor samples from solid tumor xenografts was performed as described previously previously.^27,30^ All procedures were performed in accordance with published NCRI guidelines and UK Government Home Office regulations.^58^

### Phospho-Proteome Analysis

Adherent COLO205 cells were lysed with a urea buffer, lysates were reduced with DTT, alkylated with iodoacetimide and digested with trypsin-tosylphenylalanyl chloromethyl ketone. Digested peptides were immunoprecipitated using a pool of motif antibodies from Cell Signaling Technologies (US) comprising: phospho-MAPK substrate (PXS*P or S*PXR/K, #2325, RRID:AB_331820), phospho-CDKs substrate ((K/H)­S*P, #2324; RRID:AB_2244779), phospho-PLK binding motif (ST*P, #5243; RRID:AB_10891778) and phospho-tPE motif (T*PE, #3004; RRID:AB_10890649)­of phospho-MAPK (2325), phospho-CDK (2324), phospho-PLK (5243) and phospho-tPE (C3004) motif-recognizing antibodies immobilized onto protein A/G beads. Immunoprecipitated peptides were analyzed using an LTQ-Orbitrap-Velos. Sequences were assigned to the MS/MS spectra and evaluated using SEQUEST 3G and SORCERER 2 (Sage-N Research v4.0, Milpitas). Quantitative data was evaluated and clustered in Spotfire Decision Site (www.spotfire.tibco.com) and normalized using quartile normalization. Functional annotation and pathway analysis used DAVID (https://david.ncifcrf.gov/).

### Immunoblotting and Immunoassays

Protein expression was detected and quantified using previously described immunoblotting, electrochemiluminescent assays (Mesoscale Diagnostics, US) or an automated capillary immunoassay system (Protein Simple, US).^27,30^

## Supplementary Material





## Data Availability

The mass spectrometry proteomics data have been deposited to the ProteomeXchange Consortium via the PRIDE partner repository with the data set identifier PXD061476. [https://proteomecentral.proteomexchange.org/cgi/GetDataset?ID=PXD061476]. Microarray data are available on the NCBI Gene Expression Omnibus (GEO; http://www.ncbi.nlm.nih.gov/geo/) Web site under accession number GSE67845.

## Notes

Additional experimental details on materials and methods, extended analyses of cell proliferation, phospho-proteome profiling, and gene promoter reporter assays following treatment with CDK8 inhibitors or in mediator kinase knockout cells (PDF)
Data sets related to CDK enzymatic activity assays, phospho-proteome data and corresponding analyses (data set PXD061476), and GSEA based on microarray gene expression data (data set GSE67845)^27^ (XLSX)

## §

Eli Lilly and Company, Madrid, Spain

## ∥

54Gene, Lagos, Nigeria.

## ⊥

Signaling Programme, The Babraham Institute, Cambridge, UK.

## #

Bayer, S.A.S., Lyon, France.

## ∇

The University of Texas, MD Anderson Cancer Center, Houston, Texas, USA.

## ○

NeoPhore Ltd., London, UK.
