# Supplementary material for: Mediator Kinase Inhibitor Selectivity and Activity in Colorectal Cancer
Source: ACS Chem Biol. 2025 Jul 2;20(7):1792–804. doi: 10.1021/acschembio.5c00338 (PMC12281475; doi:10.1021/acschembio.5c00338)
Supplement: Supplementary file 1 [file cb5c00338_si_001.pdf]

MEDIATOR KINASE INHIBITOR SELECTIVITY AND ACTIVITY IN COLORECTAL CANCER

Maria J. Ortiz-Ruiz, Olajumoke Popoola, Konstantinos Mitsopoulos, Robert Te-Poele, Rahul S. Samant, Gary Box, Will Court, Alexis De Haven Brandon, Sharon Gowan, Aurelie Mallinger, Toby Roe, Kate Swabey, Melanie Valenti, Bissan.Al-Lazikani, Julian Blagg, Christina Esdar, Kai Schiemann, Dirk Wienke, Suzanne A. Eccles, Paul Workman, and Paul A. Clarke

SUPPORTING INFORMATION

MATERIALS AND METHODS

CDK protein kinase assays

A radiometric protein kinase assay (^{33}PanQinase® Activity Assay, Reaction Biology, USA) was used to determine the IC_{50} profile of CDK8/19 inhibitors using the CDK-profiler service (32 human CDK:Cyclin pairs, Reaction Biology, USA). All compounds were tested at 10 semi-log assay concentrations, with 10 microM as the highest assay concentration. The final DMSO concentration in the reaction cocktails was 1 % in all cases. The residual activity (as %) for each well of a particular plate was calculated by using the following formula: Res. Activity (%) = 100 X [(cpm of compound – low control) / (high control – low control)]. The fitting model for the IC_{50} determinations was "Sigmoidal response (variable slope)" with parameters "top" fixed at 100 % and "bottom" at 0 %. The fitting method used was a least-squares fit.

Cell culture and reporter assays

Human colorectal cancer cell lines COLO205 (RRID:CVCL_0218) and SW620 (RRID:CVCL_0547) were obtained from ATCC (LGC Promochem, UK). Parent and reporter or clones were confirmed as mycoplasma-free (Lonza, Switzerland). All lines and CRIPR clones were authenticated by short tandem repeat (STR) analysis profiling using multiplex amplification of genomic loci (Penta E, D18S51, D21S11, TH01, D3S1358, FGA, TPOX, D8S1179, vWA, Amelogenin, Penta D, CSF1PO, D16S539,

D7S820, D13S317, and D5S818) with a PowerPlex 16 HS (Promega, USA). STR products were analysed on an Applied Biosystems 3500xL Genetic Analyzer (Thermo Fisher Scientific, UK) and compared to cell line reference databases.

Cells were propagated at 37 °C in a humidified incubator with 5% CO2 in air using Dulbecco's Modified Eagle's Medium (Thermo Fisher Scientific, UK) supplemented with 10% (v/v) foetal bovine serum, 2% (v/v) L-glutamine (Thermo Fisher Scientific, UK) and 1% (v/v) non-essential amino acids (Thermo Fisher Scientific, UK), and sub-cultured when they reached 80% confluence.

Stable TCF/LEF reporter cell lines

Stable SW620^{F1756} and COLO205^{F1756} TCF/LEF promoter reporter human colorectal cancer cells were generated as previously reported.[1] Briefly, cells were transduced with a lentiviral construct carrying 16 repeats of the TCF/LEF responsive element (5′-AGATCAAAGG-3′) upstream of the minimal pTA promoter driving PEST-destabilized firefly luciferase Fluc2P (t1/2 < 1 h) from plasmid pGL4.24 (Promega, USA). The TCF/LEF-pTA-Fluc2P cassette was subcloned into lentiviral vector pCDF1-MCS2-EF1-Puro (Systems Biosciences, USA) to generate the final reporter construct F1756.[1]

Clonogenic assays

Cells were seeded at 100 cells/ml of Dulbecco's modified Eagle's medium (DMEM) and 1 ml added to individual wells of a 24-well plate. After 24 hours, cells were treated with DMSO or 350 nM compounds and cultured for 10 days. Colonies were stained with 0.5% crystal violet and counted using a Gelcount colony counter (Oxford Optronix, UK).

Tumour spheroid growth kinetics and treatment with test compounds

For spheroid generation, 4000 cells were dispensed into ULA 96-well round-bottomed plates (Corning B.V. Life Sciences, The Netherlands) using a multichannel pipette. Growth kinetics and inhibition assays were performed up to 14 days after initiation. A 50% medium replenishment was performed on days 4, 7, 10 and 12 using a

multichannel pipette. Where indicated, spheroids were treated at day 4. At days 3, 6 and 8 following compound addition, 50% medium replenishment was performed. Cell-titre Glo analyses were carried out at day 14 for luminescence measurement on a Synergy 2 SL Luminescence microplate reader (BioTek, UK). Values are means ± SD (n = 6).

CRISPR/Cas9 knockout

Single and double knockout clones of human SW620 colon cancer cells lacking CDK8, CDK19 or both kinases were generated using a CRISPR/Cas9 knockout strategy. Constructs for knockout carried a Cas9 gene, a fluorescent protein gene (GFP for CDK8 and RFP for CDK19) and sgRNAs to human CDK8 (AGGACCTGTTTGAATACGAGGG) or CDK19 (AGGATTTGTTTGAGTACGAAGG). Single and double CDK8 and CDK19 knockout clones were generated by transiently transfecting SW620 cells using Lipofectamine 3000 (Thermo Fisher Scientific, UK). Cells were harvested 72 hours post-transfection and the top 1% GFP, RFP or GFP/RFP positive cells were sorted by FACS into 96 well plates at one cell/well. Knockout clones were identified by a capillary immunoassay for CDK8 or CDK19 protein expression. Stable cell lines re-expressing wild-type CDK8 or an inactive kinase mutant ($CDK8^{D173A}$)[2] were established by transfection of CDK8 CRISPR knockout clone 1 with a pCMV6-CDK8-cMyc-FLAG construct.

Reporter Panel

Transcription factor promoter reporter assays were carried out to define signalling pathways and biological processes modulated by CDK8/19 ligands. A panel of plasmid reporter constructs encoding firefly luciferase under the control of a basal promoter element with specific transcriptional response elements were used (Qiagen, Germany; **Table S1**). Firefly luciferase under the control of the minimal promoter element (TATA box) alone was used to establish the base-line signal and a construct expressing renilla luciferase from a CMV-promoter was used as a transfection control. COLO205 human colon cancer cells or SW620 parental and CRISPR KO cells were reverse-transfected with the panel of plasmid reporters in a 96 well plate format. The reporter mixes were prepared at 40:1 ratio of fLUC to rLUC plasmid at a final concentration of

100 ng/ml. For each well 1 ul plasmid was complexed with 1 ul Lipofectamine 2000 (Thermo Fisher Scientific, UK) in 50 ul OptiMEM (Thermo Fisher Scientific, UK) media for 20 mins at room temperature. Cells were trypsinised and 50 ul of cells at 2e5 cells/ml in supplemented DMEM were added to the 50 ul of plasmid:lipofectamine mixture in OptiMEM in a well of a 96-well white opaque flat bottom microtiter plate. fLUC and rLUC were measured by the addition of 100 ul dual glo luciferase reagent (Promega, USA). Reporter activity was quantified in untreated cells 48 hours post-transfection or in cells treated with CDK8/19 inhibitor at 42 hours post-transfection and assayed following a further 6 h continuous exposure to compound.

Tumour xenograft studies

The establishment, treatment, pharmacokinetic analysis and processing of tissue samples from human tumour xenografts was performed as described previously.[3,4] All procedures were performed in accordance with published guidelines and UK Government Home Office regulations.[5] Female athymic nude mice Crl:NU-*Foxn1*^{nu} (Charles River Laboratories, US) were acclimatized for 1 week prior to use. Five million SW620 human colorectal carcinoma cells were injected s.c. in the right flanks of 6–8 weeks old female NCr athymic mice under isofluorothane anesthesia. Therapy was initiated when established tumours reached a mean volume of 60 mm (day 7). Animals were randomly assigned into cohorts. Control mice received vehicle (0.5% Methocel, 0.25% Tween 20 in sterile phosphate buffered saline and treated animals CCT251921 at 42 mg/kg p.o qd for 5/7 days with a 2-day break after 1 week's dosing. CCT251921 was prepared 24 h prior to administration to mice by weighing into a glass 30 mL universal tube, and Methocel solution (0.5% Methocel, 0.25% Tween 20 in sterile phosphate buffered saline) added to provide a final concentration of 3 mg/mL. The preparation was agitated at room temperature by magnetic stirrer to bring it into a fine suspension before use. Animals were dosed orally by gavage every 24 h at 0.1 mL per 10 g body weight. Tumours were measured three times weekly by Vernier calipers and body weights recorded. At the end of the study, day 3 for pharmacodynamic biomarker analysis and day 14 for anti-tumour therapy experiments, animals were culled 6 h after the final dose. Heparinized blood was collected by cardiac puncture, spun, and the plasma snap frozen in liquid nitrogen for analysis of compound exposure. Tumours were excised, weighed and samples snap frozen for compound

quantification and pharmacodynamic biomarker analyses. All samples were stored −80 °C.

Pharmacokinetic analysis

Plasma homogenates were extracted with 3 equivalent volumes of methanol containing olomoucine (500 nM) as an internal standard. Extracts were quantified for compound CCT251921 using an external calibration method (8-point calibration curve ranging from 2 nM to 50 000 nM) with 4 quality control samples (25, 250,2500, and 7500 nM in duplicate) included at the beginning and the end of the analytical run. Plasma homogenates were measured by LC−MS/MS on a Waters TQS following a separation on a Waters Acquity BEH C18 column (50 mm × 2.1 mm; 1.7 μm) with conditions of 0.1% formic acid (mobile phase A) and methanol (mobile phase B). The column was equilibrated at initial condition of 95% A and 5% B, linear gradient over 3 min to 100% B, held over 1 min, followed by linear gradient back to 5% B over 0.1 min, at 0.6 mL/min flow rate. Detection was achieved in positive electrospray ionization mode by multiple reaction monitoring, 412.17 > 376.40at 33 eV for compound 109, and 299.19 > 91.24 at 33 eV, 299.19 >177.29 at 26 eV for the internal standard olomoucine.

Phospho-proteome analyses

Adherent COLO205 cells were washed with PBS and cells lysed using urea lysis buffer (9 M Urea, 20 mM HEPES, pH 8.0, 1 mM beta-glycerophosphate, 1 mM sodium vanadate, 2.5 mM sodium pyrophosphate) and stored at -80 ^{o}C prior to analysis. The supernatants were reduced with 100 mM DTT for 30 minutes at 55 ^{o}C, followed by alkylation with 50 mM iodoacetimide for 15 minutes at ambient temperature in the dark. The samples were diluted in 20 mM HEPES, pH 8.0 and digested overnight with 10 ug/mL trypsin-tosylphenylalanyl chloromethyl ketone in 1 mM HCl. Digested peptides were acidified with 1% formic acid and then desalted over 360-mg SEP PAK C18 columns (Waters, UK). Peptides were eluted with 40% acetonitrile in 0.125% formic acid, dried under a vacuum and the lyophilised peptides used for immunoprecipitation.

A pool of motif antibodies from Cell Signaling Technologies (US) comprising: phospho-MAPK substrate (PXS*P or S*PXR/K, #2325, RRID:AB_331820), phospho-CDKs substrate ((K/H)S*P, #2324; RRID:AB_2244779), phospho-PLK binding motif (ST*P, #5243; RRID:AB_10891778) and phospho-tPE motif (T*PE, #3004; RRID:AB_10890649), were immobilised onto protein A/G beads overnight at 4 ^{o}C. The lyophilised peptides were resuspended in MOPS buffer (50 mM MOPS, pH 7.2, 10 mM KH_2PO_4, 50 mM NaCl) and centrifuged for 5 mins at 13,500 rpm in an Eppendorf benchtop microcentrifuge. The supernatants were incubated with the motif-bound beads for 2.5 hours at 4 ^{o}C . The beads were harvested by centrifugation at 4000 rpm in an Eppendorf benchtop microcentrifuge for 5 mins, washed with MOPS buffer twice and washed again 5 times with deionised water. The peptides were eluted off the beads using 0.125% formic acid, were desalted and dried under a vacuum. The lyophilised immunoprecipitated peptides were resuspended in 0.125% formic acid and separated on a capillary column packed with Magic C18 AQ reversed-phase resin. Peptides were eluted using a 72-minute linear gradient of acetonitrile in 0.125% formic acid delivered at 280nL/min. Samples were analysed using an LTQ-Orbitrap-Velos. MS Parameter Settings were: MS Run Time 96 min, MS1 Scan Range (300.0 – 1500.00), Top 20 MS/MS (Min Signal 500, Isolation Width 2.0, Normalized Coll. Energy 35.0, Activation-Q 0.250, Activation Time 20.0, Lock Mass 371.101237, Charge State Rejection Enabled, Charge State 1+ Rejected, Dynamic Exclusion Enabled, Repeat Count 1, Repeat Duration 35.0, Exclusion List Size 500, Exclusion Duration 40.0, Exclusion Mass Width Relative to Mass, Exclusion Mass Width 10ppm). Sequences were assigned to the MS/MS spectra and evaluated using SEQUEST 3G and the SORCERER 2 platform (Sage-N Research v4.0, US). Searches were performed against the NCBI *homo sapiens* database (GRCh37/hg19) with mass accuracy of +/- 50 ppm for precursor ions and 1 Da for product ions. Results were filtered with mass accuracy of +/- 5 ppm on precursor ions and presence of the intended motif. A 5% default false positive rate was used to filter the SORCERER results. Quantitative data was evaluated and clustered in Spotfire Decision Site (www.spotfire.tibco.com) and normalized using quartile normalization. Functional annotation and pathway analysis used DAVID (https://david.ncifcrf.gov/).

Immunoprecipitation

Cell monolayers were washed with PBS twice and lysed using a modified RIPA buffer, mRIPA (50 mM TRIS-HCl pH 7.4, 1% NP-40 substitute, 0.5% NaDOC, 150 mM NaCl, 1 mM EDTA, 0.02% SDS and 1:100 dilution of proteinase and phosphatase inhibitor cocktail, (#5872, Cell Signaling Technology) with overnight freezing at -80 °C and sonication of the cell pellet. Lysates were clarified by centrifugation and supernatants collected and protein concentration determined by a bicinchoninic acid (BCA) assay. One mg of total protein was diluted in 400 μL of mRIPA buffer and incubated with a cocktail of phospho-motif antibodies, corresponding to those used in the phospho-proteome analyses, MAPK substrate (#2325) at 1.8 ug + phospho-T*PE (#3004) at 1.7 μg + phospho-PLK binding motif (#5243) at 0.5 μg + CDKs substrate (#2324) at 9.7 μg) for 24 hours at 4 °C. Antibody-antigen complexes were recovered using Protein G beads and the antibody-protein-bead complexes washed 10 times with mRIPA buffer. The beads were resuspended in Laemli sample buffer containing DTT reducing agent and the complexes resolved by SDS-PAGE and immunoblotting.

Protein detection by immunoassays

Tissue culture cells were washed with PBS and lysed in ice-cold lysis buffer (140 mM NaCl, 10 mM EDTA, 10 % glycerol, 1 % Nonidet P-40, 20 mM Tris (pH 8.0), 1 mM pepstatin, aprotinin at 1 μg/ml, leupeptin at 1 μg/ml, 1 mM phenylmethylsulfonyl fluoride, 1 mM sodium orthovanadate), and frozen at −80 °C overnight. After the cell lysates were thawed, samples were centrifuged at 10,000 × g at 4°C for 10 min and supernatants were transferred to new tubes and protein concentration measured using a BCA assay.

Excised tumour samples were transferred to MK28 reinforced homogenizing tubes with metal beads (Stretton Scientific, UK) and lysis buffer (50 mM Tris-HCl, pH7.4, 1 mM EDTA, 1% Triton X-100(v/v), 150 mM NaCl, 1 mM activated sodium orthovanandate, 1 mM PMSF, protease cocktail (1:100 P8340; Sigma UK), and phosphatase inhibitors (1:50 dilution, P5726 and P0044; Sigma, UK) were added immediately. The samples were ground using a Precellys 24 set at 6,000 rpm, 2 × 20 sec (Stretton Scientific, UK). The tumour lysates were put through a freeze-thaw cycle at −80 °C, sonicated using in sonicating water bath at room temperature for 3 min and then incubated on ice for 10 min. Protein supernatants were recovered following

centrifugation in an Eppendorf benchtop microcentrifuge pre-chilled to 4 °C at top speed (14000 rpm) for 10 min. Tumour lysates were diluted 1:5 in lysis buffer and protein concentrations determined using a Direct Detect spectrometer (Merck Millipore, UK). Supernatants were then aliquoted and frozen at −80 °C until analysis.

Protein expression was detected and quantified using previously described immunoblotting, electrochemiluminescent assays (Mesoscale Diagnostics, US) or an automated capillary immunoassay system (Protein Simple, US).[6] Antibodies were obtained from Cell Signaling Technologies (CDK8, #4106, RRID:AB_1903936; CHD4, #11921; EGR1, #4154; p-STAT1^{SER727}, #8826 and #9177; Vinculin, #11912; B-actin, #4970, RRID:AB_2223172), Bethyl Laboratories (cyclin C, A301-989A, RRID:AB_1576505; pRNA pol II CTD^{SER2}, A304-407A, RRID:AB_2631452; pRNA pol II^{SER5}, A304-408A, RRID:AB_2620602; total RNA pol II, A300-653A, RRID:AB_519334), Abcam (Med13, Ab76923, RRID:AB_1523933; MED13L, Ab87831, RRID:AB_2043038; MED14, Ab72141, RRID:AB_1209425; GAPDH, Ab8245, RRID:AB_2107448), Santa Cruz (Med12, #5372, RRID:AB_2235318; total STAT1, #346, RRID:AB_632435) Millipore (HCFC1, abd74) and Sigma-Aldrich (CDK19, HPA007053, RRID:AB_1233803). Antibodies for pE2F1^{SER375} and total-E2F1 were generated as described previously.[3]

For polyacrylamide gel separation, samples were aliquoted, then boiled in electrophoresis sample buffer, loaded and separated on SDS-4-12% PAGE gels. After electro-transfer to PVDF membranes, blots were blocked at room temperature for 1 h in blocking buffer (5% dry milk in TNT: 1 M Tris-HCL, pH 8, 5 M NaCl, 0.1% Tween 20) and then incubated at 4°C overnight with the appropriate antibody. After washing with TNT, blots were incubated with HRP-conjugated secondary antibodies in blocking buffer at room temperature for 1 h, and signal were visualized using the enhanced chemoluminescence (ECL prime) method.

Detection plates for the electrochemiluminescent assays were prepared by diluting STAT1 (Cell Signaling Technologies, #9176, RRID:AB_2240087) antibody stock to 2 µg/ml or 1 µg/ml in PBS, for phospho- or total-STAT1 assays respectively and adding 25 µl per well to a standard bind plate (L15XA-3, Mesoscale Diagnostics, US). The plate was gently tapped until equal coverage of each well was achieved. The plate

was incubated overnight at 4°C. Contents were tapped out and 150 µl per well of 3 % blocking solution (600 µg Blocker A - R93BA - Mesoscale Diagnostics, US - in 20 mL wash buffer plus 500 µl BSA solution) was added. Plates were sealed and shaken on a plate shaker for 1 hour, then washed 3 times with 150 µl per well of wash buffer using a BioTek ELx50 plate washer. 25 µl per well diluted lysate was added and incubated for overnight at 4°C. Plates were washed 3 times with 150µl per well of wash buffer (50 mL 1M Tris-HCl pH 8.0, 30 mL 5M sodium chloride, 1 mL Tween 20 made up to 1 L with deionised water). 25 µl of 2 µg/ml or for phospho (ab47754) or total (Cell Signaling Technology, #9172, RRID:AB_2198300) STAT1 respectively diluted in 1 % BSA in wash buffer per ml was plated and placed on a plate shaker for 1 h. Plates were washed 3 times as before. 25 µl per well of sulpho-tag anti-rabbit antibody diluted to 2 µg/ml in 1 % BSA was added and incubated for 1 h on shaker. Plates were washed 3 times as before. 150 µl per well of 2 x read buffer (Mesoscale Diagnostics, US) was added immediately prior to reading the plate on a Quickplex 120 (Mesoscale Diagnostics, US).

Capillary immunoassays were performed according to the manufacturer's instructions. Briefly, protein lysates were diluted to a final concentration of 0.4 mg/ml and 4 µl loaded onto the 12-230 kDa (Bio-Techne cat: SM-W004) assay plate. Primary antibodies were diluted as follows: CDK8 1:25; CDK19 1:50; p-STAT 1:50; STAT 1:100 and β-actin 1:100. Secondary antibodies were used from the detection packs (Protein Simple cat no: DM-001 or DM-002 for anti-rabbit or mouse respectively). Standard default run conditions were used with a 25 min separation time, separation voltage 375, antibody diluent time 5 min and antibody incubations 30 min each. Data was analysed using Compass for Simple Western software.

SUPPORTING TABLES AND LEGENDS

Transcription factor	Pathway	Transcription factor	Pathway
AARE	ATF2/3/4 Amino acid deprivation	MEF2	MEF2
AR	Androgen	MTF1	Heavy metal
ARE	Nrf1/Nrf2/Antioxidant response	Myc	cMYC
AP1	MAP/JNK/FOS/EGR/ATF	Nanog	Nanog
ATF6	ATF6	NFAT	PKC/Ca2+
C/EBP	C/EBP	NFkB	NFkB
CRE	CREB/cAMP/PKA	OCT4	OCT4
E2F	Cell cycle	p53	DNA damage
EGR1	EGR1	Pax6	Pax6
ERE	ER/Estrogen	PPAR	PPAR
ERSE	CBF/NF-Y/YY1/Endoplasmic reticulum stress	PR	Progesterone
FOXO	PI3K/Akt	RARE	RAR/Retinoic acid
GAS	STAT1/Interferon gamma	RBP-Jk	Notch
GATA	GATA	RXR	Retinoid X
GLI	Hedgehog	SMAD	SMAD2/3/4/TGF-β
GRE	GR/Glucocorticoid	Sox2	Sox2
HIF	HIF-1α/Hypoxia	SP1	SP1
HNF4	HNF4	SRE	SRF/ELK1/MAPK/Erk
HSR	HSF1/Heat shock	STAT3	STAT3
IRF1	Interferon regulation	TCF/LEF	WNT
ISRE	STAT1/STAT2/Type I interferon	VDR	Vitamin D
KLF4	KLF4	XRE	AhR/Xenobiotic
LXR	Liver X		

Supporting Table S1. Summary of transcription factors and pathways assayed in the promoter reporter panel.

SUPPORTING FIGURES AND LEGENDS

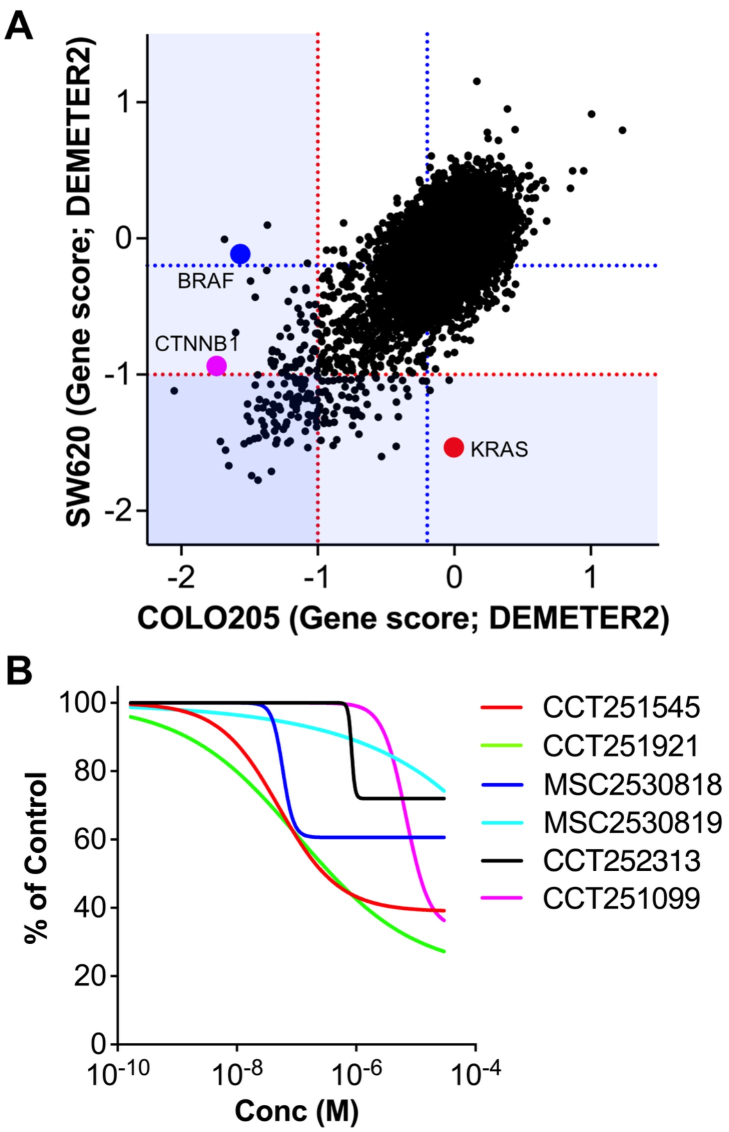

Supporting Figure S1. Effect of CDK8/19 inhibition on human colorectal cancer cells with different dependencies on KRAS or Beta-catenin. **(A)** shRNA DEPMAP gene scores (DEMETER2) for COLO205 or SW620 cells highlight their dependencies on beta-catenin (CTNNB1) or KRAS/BRAF/MAPK signaling pathways. The blue dotted line indicates the threshold for a knockdown having a minimal effect on cell survival and/proliferation, and the red dotted line indicate a threshold for the knockout effecting cell proliferation and/or survival. **(B)** Linear regression plots show the response of SW620 colorectal cancer cells (n = 2 independent repeats) expressing a TCF/LEF WNT-reporter construct (F1756) following 6 hours exposure to compound. Data are fitted using a log(inhibitor) versus response model with a four-parameter variable slope; symbols and error bars are omitted for simplicity.

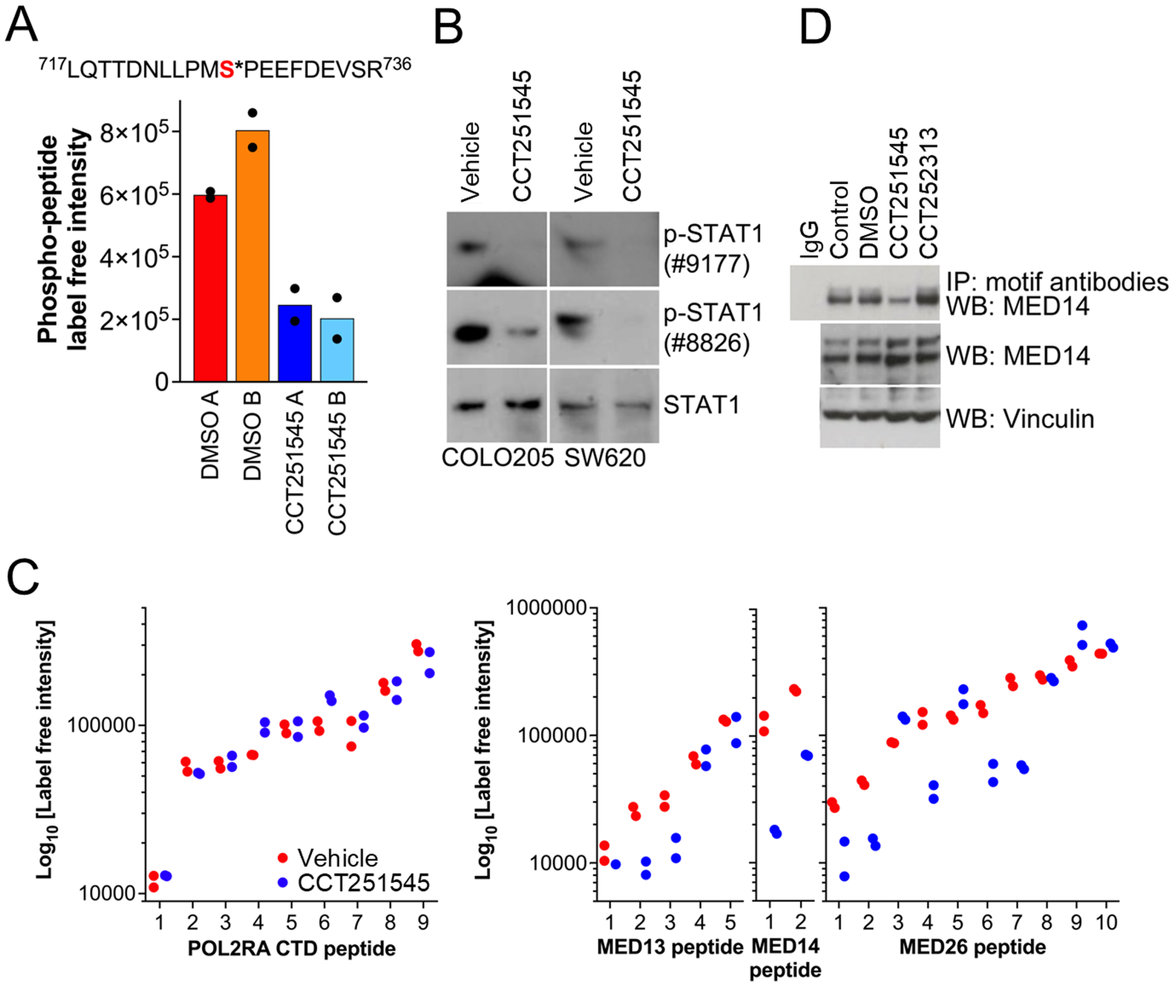

Supporting Figure S2. Protein phosphorylation identified by phospho-proteome profiling. (A) Label-free intensity of the STAT1 phospho-peptide ^{717}LQTTDNLLPMS*PEEFDEVSR^{735} in vehicle- and CCT251545-treated samples (A/B = biological repeats; * = phosphorylated residue). **(B)** STAT1^{SER727} phosphorylation in COLO205 or SW620 cell lysates detected using two different phospho-STAT1^{SER727}-specific antibodies following treatment with 350 nM CCT251545 for 6 hours. Total STAT1 served as a loading control. **(C)** Label-free intensities of individual phospho-peptides from the RNA Pol II CTD, MED13, MED14 and MED26 (n = 2 independent repeats). **(D)** Immunoprecipitation of protein from COLO205 cell lysates following treatment with 350 nM compounds for 6 hours with CDK-motif-specific antibodies, followed by immunoblotting for MED14, with Vinculin measured in lysates as a loading control.

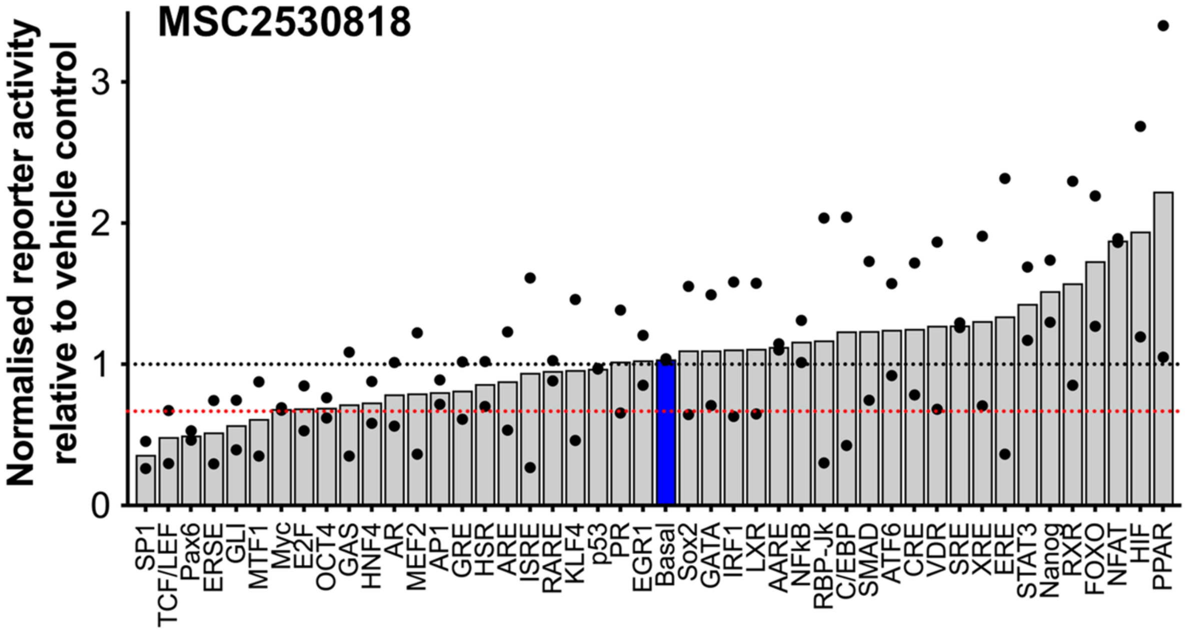

Supporting Figure S3. Promoter reporter activity following treatment of COLO205 cells with CDK8/19 inhibitor MSC2530818. Promoter reporter assay in COLO205 cells treated with 350 nM MSC2530818, for 6 hours (equivalent to 10x EC_{50} for TCF/LEF activity in the COLO205-cl4 reporter line; n = 2 independent repeats). The blue column indicates the basal transcriptional reporter activity.

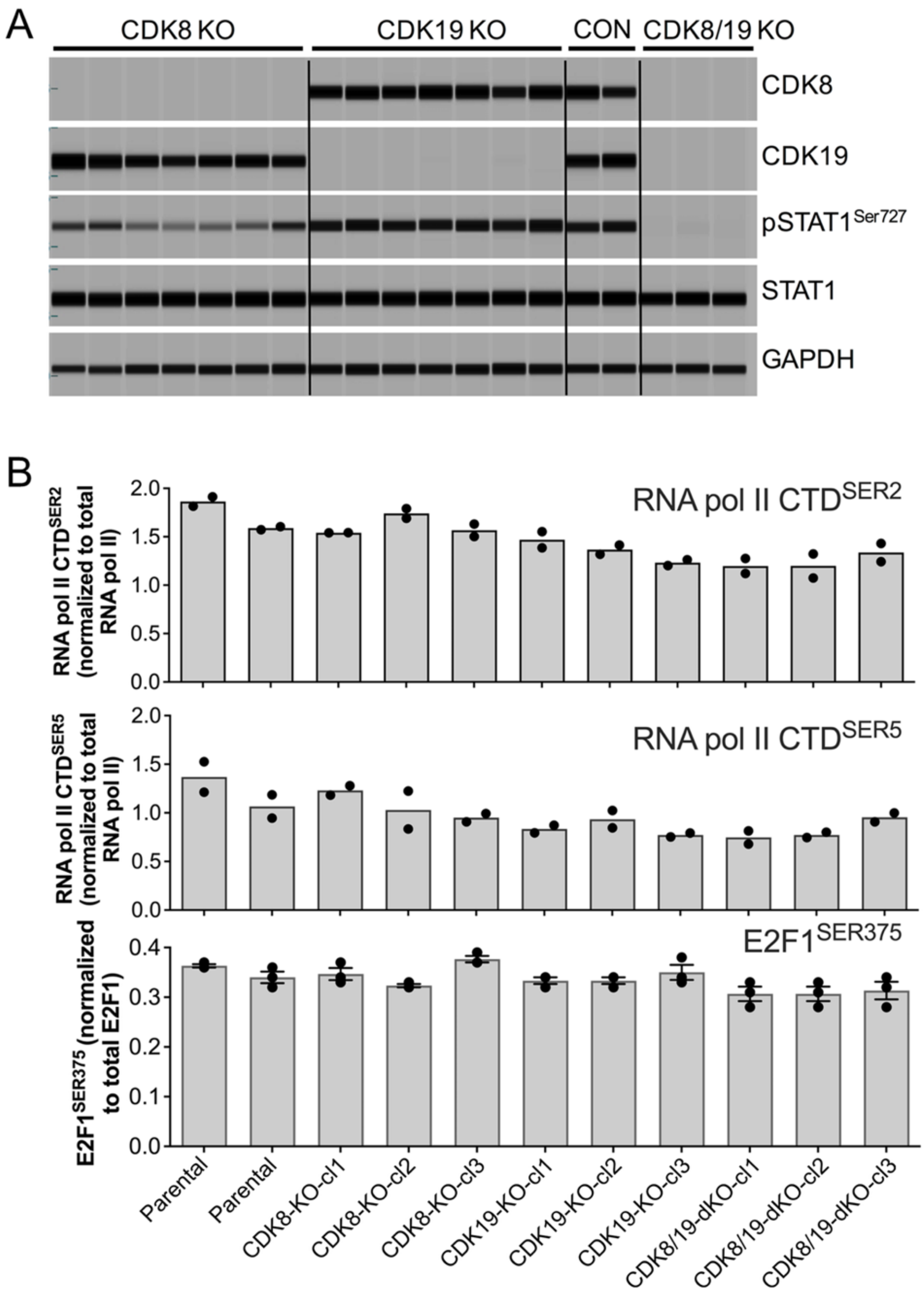

Supporting Figure S4. Generation of CDK8 and/or CDK19 knockout lines by CRISPR/Cas9 technology in SW620 human colon cancer cells. (A) Capillary immunoassay of CDK8, CDK19 and phospho-STAT1^{SER727} in lysates of multiple CRISPR/CAS9 clones selected for evidence of CDK8 and/or CDK19 loss. **(B)** Capillary immunoassay of independent repeats of phosphorylation of reported CDK8 substrates RNA pol II^{SER2},RNA pol II^{SER5} (n = 2) and pE2F1^{SER375} (n = 3) in selected SW620 parent or CRISPR/Cas9 knockout clones.[6–8]

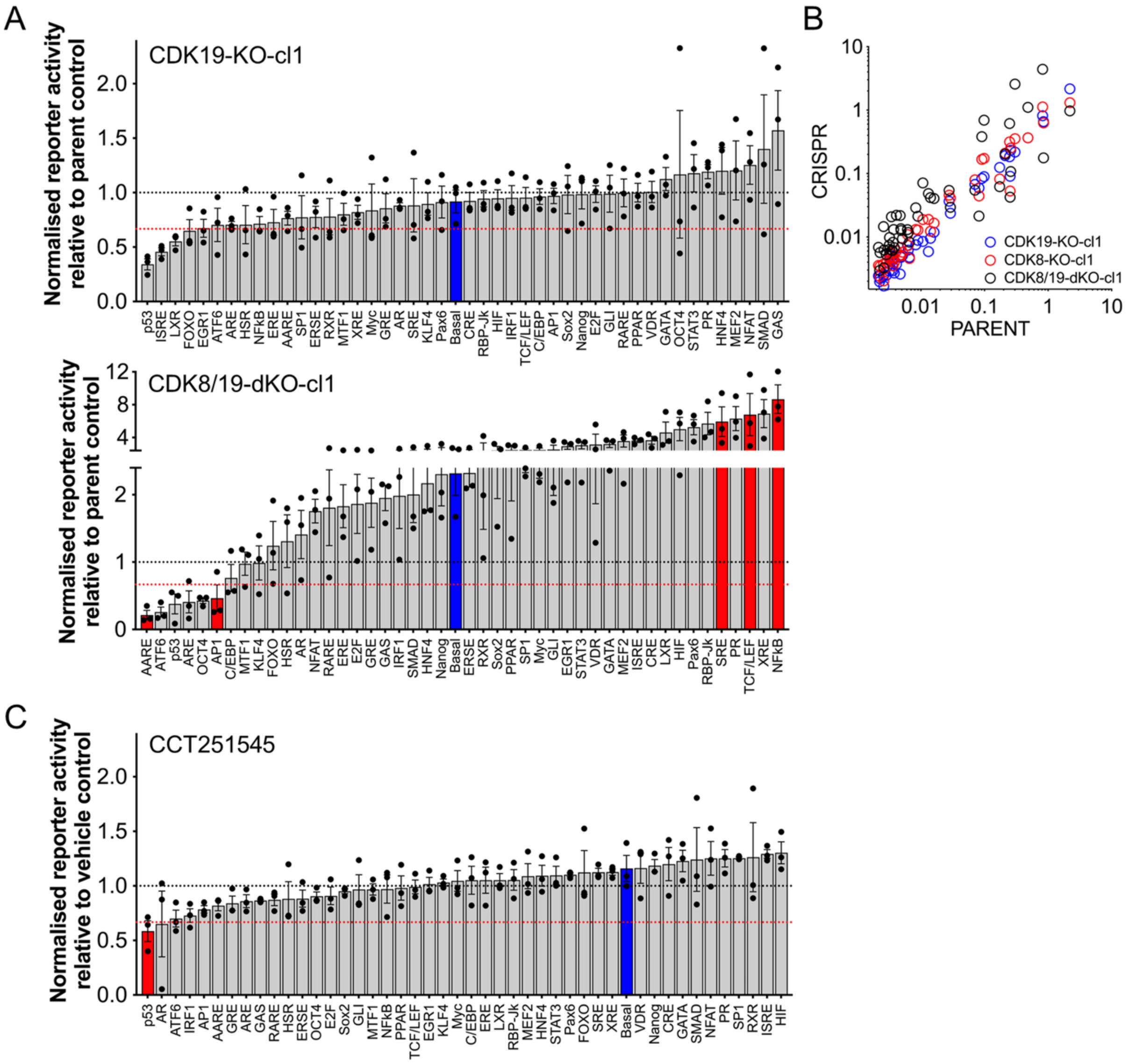

Figure S5. Promoter reporter activity in CDK19 or CDK8/19 knockout SW620 human colorectal cancer cells. **(A)** Plot of transcription factor promoter reporter array results depicting the impact of CDK19 or CDK8/19 double knockout relative to parent SW620 cells. **(B)** Summary plot comparing promoter reporter data from CRISPR knockout models versus the parent SW620 cells. Each symbol represents the ratio of the individual promoter reporter activity relative to the co-transfected control CMV promotor reporter used for normalisation. **(C)** Plot of transcription factor promoter reporter array results depicting the impact of 6 hours treatment of parent SW620 cells with 350 nM CCT251545. For **(A)** and **(C)** data represent mean ± s.e.m (n = 3). The black line indicates unchanged reporter activity, while the red line highlights reporters with altered ± 1.5-fold. The blue column represents basal transcriptional reporter

activity, and the red column indicates significance ($p_{adj} < 0.001$; ANOVA with Sidak's correction for multiple testing).

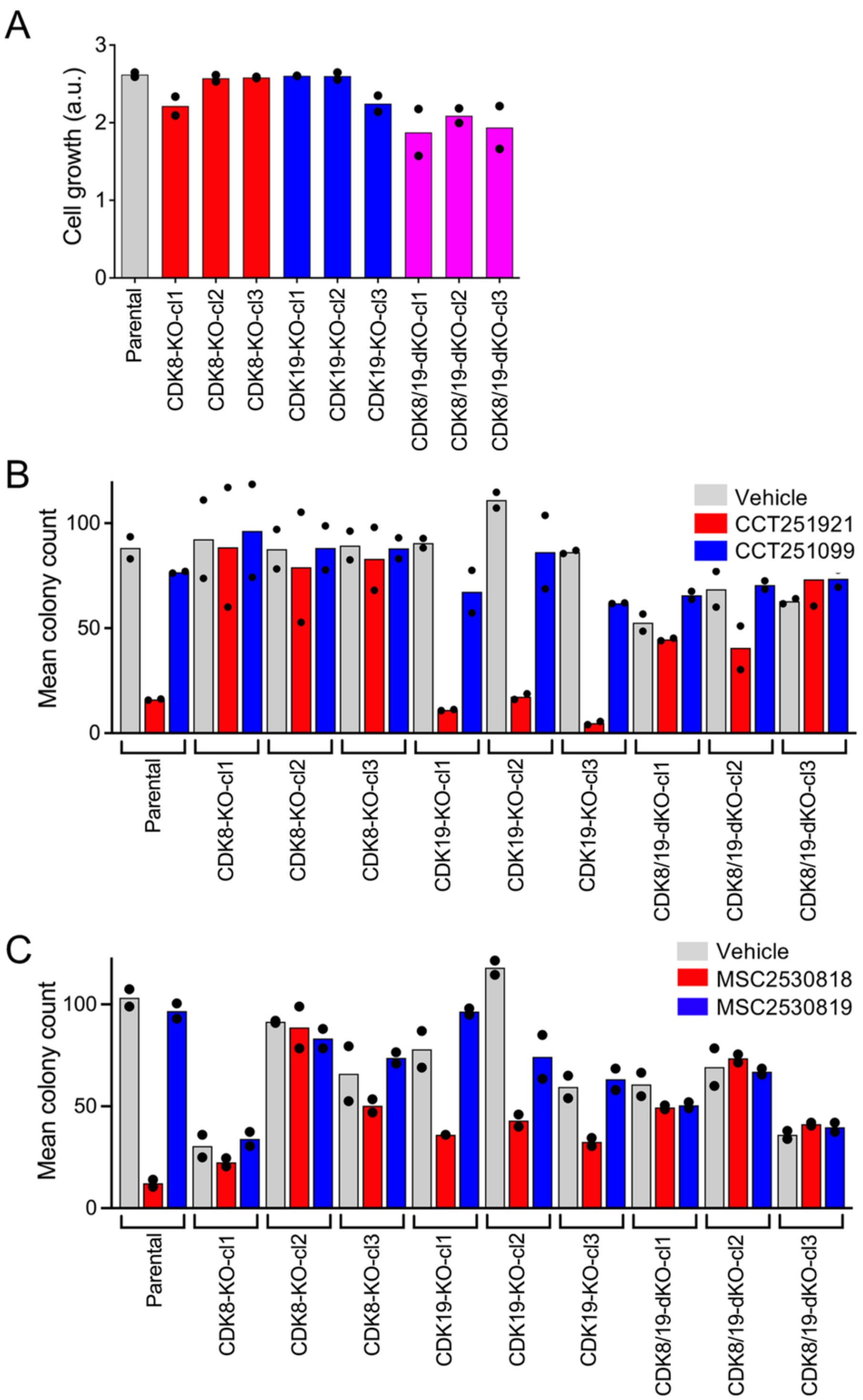

Supporting Figure S6. Loss of CDK8 abrogates growth inhibition following treatment of SW620 human colorectal cancer adherent colony assays with CDK8/19 inhibitors. (**A**) Alamar Blue analysis of adherent growth was performed on untreated SW620 parent cells and knockout clones at 96 hours (n = 2). (**B**) Colony

growth assay of SW620 parent cells and knockout clones was conducted following 10 days of treatment with either 350 nM CCT251921 or CCT251099, a structurally related but less active control compound (n =2). (**C**) Similar assays were performed with 350 nM MSC2530818, or MSC2530819 a structurally related but less active control (n = 2).

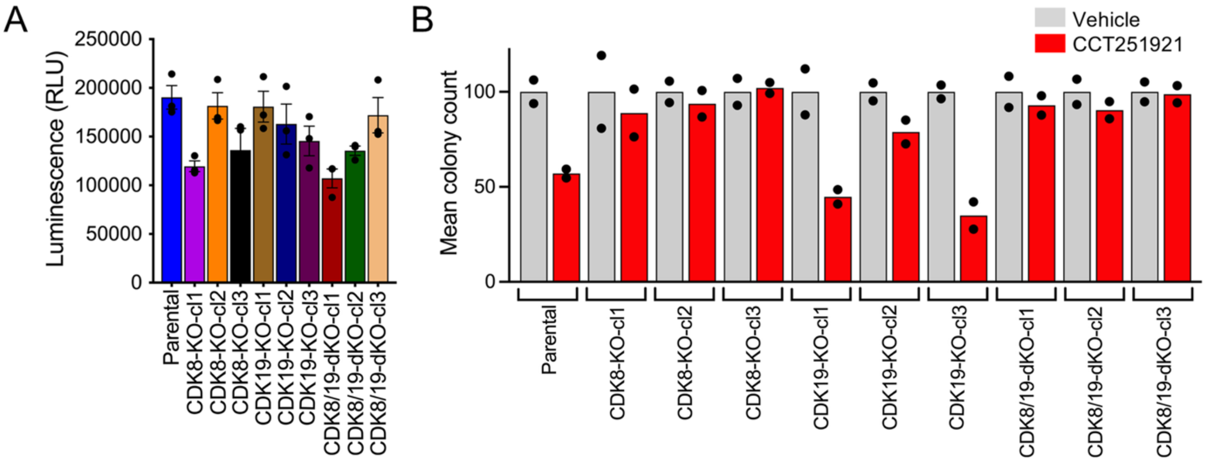

Supporting Figure S7. Loss of CDK8 abrogates growth inhibition following treatment of SW620 human colorectal cancer spheroid cultures with a CDK8/19 inhibitor. (A) CellTitre-Glo analysis was used to assess the 3D tumour spheroid growth of untreated SW620 parent cells or knockout clones (mean ± s.e.m, n = 3). (B)Tumour spheroid growth was analyzed after 14 days of treatment with 350 nM CCT251921 (mean, n=2).

REFERENCES

(1) Mallinger, A.; Crumpler, S.; Pichowicz, M.; Waalboer, D.; Stubbs, M.; Adeniji-Popoola, O.; Wood, B.; Smith, E.; Thai, C.; Henley, A. T.; Georgi, K.; Court, W.; Hobbs, S.; Box, G.; Ortiz-Ruiz, M.-J.; Valenti, M.; De Haven Brandon, A.; TePoele, R.; Leuthner, B.; Workman, P.; Aherne, W.; Poeschke, O.; Dale, T.; Wienke, D.; Esdar, C.; Rohdich, F.; Raynaud, F.; Clarke, P. A.; Eccles, S. A.; Stieber, F.; Schiemann, K.; Blagg, J. Discovery of Potent, Orally Bioavailable, Small-Molecule Inhibitors of WNT Signaling from a Cell-Based Pathway Screen. *Journal of Medicinal Chemistry* **2015**, *58* (4), 1717–1735. https://doi.org/10.1021/jm501436m.

(2) Barette, C.; Jariel-Encontre, I.; Piechaczyk, M.; Piette, J. Human Cyclin C Protein Is Stabilized by Its Associated Kinase Cdk8, Independently of Its Catalytic Activity. *Oncogene* **2001**, *20* (5), 551–562. https://doi.org/10.1038/sj.onc.1204129.

(3) Dale, T.; Clarke, P. A.; Esdar, C.; Waalboer, D.; Adeniji-Popoola, O.; Ortiz-Ruiz, M. J.; Mallinger, A.; Samant, R. S.; Czodrowski, P.; Musil, D.; Schwarz, D.; Schneider, K.; Stubbs, M.; Ewan, K.; Fraser, E.; TePoele, R.; Court, W.; Box, G.; Valenti, M.; De Haven Brandon, A.; Gowan, S.; Rohdich, F.; Raynaud, F.; Schneider, R.; Poeschke, O.; Blaukat, A.; Workman, P.; Schiemann, K.; Eccles, S. A.; Wienke, D.; Blagg, J. A Selective Chemical Probe for Exploring the Role of CDK8 and CDK19 in Human Disease. *Nature Chemical Biology* **2015**, *11* (12), 973–980. https://doi.org/10.1038/nchembio.1952.

(4) Clarke, P. A.; Ortiz-Ruiz, M.-J.; TePoele, R.; Adeniji-Popoola, O.; Box, G.; Court, W.; Czasch, S.; El Bawab, S.; Esdar, C.; Ewan, K.; Gowan, S.; De Haven Brandon, A.; Hewitt, P.; Hobbs, S. M.; Kaufmann, W.; Mallinger, A.; Raynaud, F.; Roe, T.; Rohdich, F.; Schiemann, K.; Simon, S.; Schneider, R.; Valenti, M.; Weigt, S.; Blagg, J.; Blaukat, A.; Dale, T. C.; Eccles, S. A.; Hecht, S.; Urbahns, K.; Workman, P.; Wienke, D. Assessing the Mechanism and Therapeutic Potential of Modulators of the Human Mediator Complex-Associated Protein Kinases. *eLife* **2016**, *5*, e20722. https://doi.org/10.7554/eLife.20722.

(5) Workman, P.; Aboagye, E. O.; Balkwill, F.; Balmain, A.; Bruder, G.; Chaplin, D. J.; Double, J. A.; Everitt, J.; Farningham, D. A. H.; Glennie, M. J.; Kelland, L. R.; Robinson, V.; Stratford, I. J.; Tozer, G. M.; Watson, S.; Wedge, S. R.; Eccles, S.

A.; Navaratnam, V.; Ryder, S. Guidelines for the Welfare and Use of Animals in Cancer Research. *Br.J.Cancer*, 2010, *102*, 1555–1577. https://doi.org/10.1038/sj.bjc.6605642.

(6) Rickert, P.; Corden, J. L.; Lees, E. Cyclin C/CDK8 and Cyclin H/CDK7/P36 Are Biochemically Distinct CTD Kinases. *Oncogene* **1999**, *18* (4), 1093–1102. https://doi.org/10.1038/sj.onc.1202399.

(7) Zhao, J.; Ramos, R.; Demma, M. CDK8 Regulates E2F1 Transcriptional Activity through S375 Phosphorylation. *Oncogene* **2013**, *32* (30), 3520–3530. https://doi.org/10.1038/onc.2012.364.

(8) Whittaker, S. R.; Mallinger, A.; Workman, P.; Clarke, P. A. Inhibitors of Cyclin-Dependent Kinases as Cancer Therapeutics. *Pharmacology & Therapeutics* **2017**, *173*, 83–105. https://doi.org/10.1016/j.pharmthera.2017.02.008.
